# Recruiting at the Edge: Kinetic Energy Inhibits Anchovy Populations in the Western Mediterranean

**DOI:** 10.1371/journal.pone.0055523

**Published:** 2013-02-25

**Authors:** Javier Ruiz, Diego Macías, Margarita M. Rincón, Ananda Pascual, Ignacio A. Catalán, Gabriel Navarro

**Affiliations:** 1 Department of Coastal Ecology and Management, Instituto de Ciencias Marinas de Andalucía, Consejo Superior de Investigaciones Científicas, Campus Río San Pedro, Puerto Real, Cádiz, Spain; 2 Institut Mediterrani d'Estudis Avançats (IMEDEA-CSIC/UIB), Esporles, Balearic Islands, Spain; Aristotle University of Thessaloniki, Greece

## Abstract

The Strait of Gibraltar replenishes the Mediterranean with Atlantic waters through an intense eastward current known as the Atlantic Jet (AJ). The AJ fertilizes the southwestern Mediterranean and is considered to be the ultimate factor responsible for the comparatively high fish production of this region. Here, we perform an analysis of the available historical catches and catch per unit effort (CPUE), together with a long series of surface currents, kinetic energy and chlorophyll concentration. We show that the high kinetic energy of the AJ increases primary production but also negatively impacts the recruitment of anchovy. We contend that anchovy recruitment in the region is inhibited by the advection and dispersion of larvae and post-larvae during periods of strong advection by the AJ. The inhibitory impact of kinetic energy on anchovy landings is not a transient but rather a persistent state of the system. An exceptional combination of events creates an outbreak of this species in the Alboran Sea. These events depend on the Mediterranean-Atlantic exchange of water masses and, therefore, are highly sensitive to climate changes that are projected, though not always negatively, for fish landings.

## Introduction

The Alboran Sea (Figure S1) is the door to replenish with Atlantic waters the Mediterranean basin, where evaporation rates and deep outflow cannot be compensated by river discharges and atmospheric precipitations. Replenishment occurs via an intense eastward current, commonly named the Atlantic Jet (AJ), through the Strait of Gibraltar. The persistence and energy of the AJ, with standard velocities of 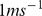, drive the circulation of the Alboran Sea [1]. The climatological features of this basin typically include a western and an eastern anticyclonic gyre (WAG and EAG, respectively; see Figure S1), but circulation snapshots with one, three or even no gyres are also common [2]–[4]. This diversity emerges from changes in the intensity and direction of the AJ that are driven by seasonal oscillations [5], by variations in the atmospheric pressure of the western Mediterranean [6] and by the wind stress in the Strait of Gibraltar [7].

Circulation instabilities manifest as complex meso- and submesoscale processes that fertilize the surface waters of the basin [8]. Positive vorticity enhances the production in offshore waters between WAG and EAG [9]. Cyclonic circulation forced by the AJ at the northwestern shelf of the Alboran Sea also increases the production of this area, particularly under westerly winds that strengthen the jet [10] and trigger coastal upwelling [11]. Vertical dynamics at the WAG edge [12], [13] and horizontal advection from the shelf or even from the Atlantic side of the strait [8], [14], [15] create a strip of high chlorophyll levels following the AJ around the anticyclonic gyres. All together, these mechanisms make the Alboran Sea a productive sub-basin [16] that eludes the severe oligotrophy that characterizes the Mediterranean [17].

The elevated primary production of the Alboran Sea should result in potential fish landings that are comparatively high when contrasted to the oligotrophic Mediterranean, particularly for small pelagic species [18]. Due to its economic value, European anchovy (*Engraulis encrasicolus*) has been the main target species for purse seines since the late 1960 s [19]. Similar to other anchovy fisheries [20], landings in the Alboran Sea are mainly based on age-0 recruits and have strong interannual variability [19]. However, major freshwater inputs known to force anchovy catches at the nearby Gulf of Cadiz [20], the Bay of Biscay [21] and the Catalan Sea [22] are absent in the Alboran basin, and the origin of landing fluctuations has remained largely unknown.

The fish in this area have most likely adapted to the prevailing hydrodynamic regime and narrow shelf. In the Alboran Sea, anchovy spawn from late spring to early autumn at 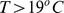, and recruits enter the fishery in the same year [23], [24]. Several authors identify the protection from intense currents provided by Malaga Bay as being an important factor for spawning and nursery grounds for small pelagic species in the north Alboran Sea [25]–[29]. However, these areas are strongly affected in their southern regions by the AJ, which alters the composition of the ichthyoplanktonic fish assemblage by transporting early stages away from the coast into the highly dynamic Alboran Sea and is able to transport mesopelagic larvae inshore via vertical upwelling [30]. It is expected that the AJ dynamics, including north-south excursions, will have an impact on the subsequent recruitment of species, particularly on small pelagic, which are known to be very sensitive to the physical environment [31]. However, the available knowledge on the environmental control of anchovy recruitment in the area, which seems largely unconnected to the spawning stock biomass in the last decades [24], remains speculative and based upon short-term analyses of growth and/or condition [27].

In this paper, we perform an extensive analysis based on the joint signals provided by available historical fisheries records and data from remote sensors for surface currents, kinetic energy (KE), temperature and chlorophyll. Based on this analysis, we conclude that anchovy recruitment in the Alboran Sea is highly linked to the dynamics of the AJ and the associated circulation features in the basin. An anomalous environment results in success rather than failure in recruitment. The standard state of the basin is highly energetic because of the jet. This jet enhances primary production but consistently creates adverse conditions because it advects and disperses the early life-stages of anchovy. Anomalous years with lower levels of kinetic energy result in high recruitment and subsequent landings.

## Results

**Figure 1:**
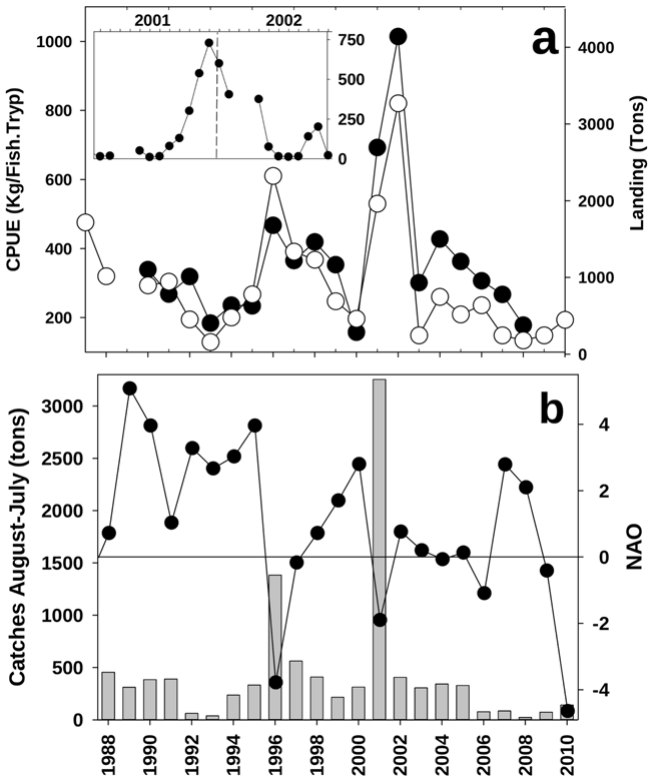
Anchovy fishery at the Alboran Sea. a) White and black circles are landings and CPUE respectively. The insert shows monthly landing (Tons) at the period of maximum catches. b) Bars show the accumulated catches between August and July of the next year (assigned to the first of the two years involved) and circles the value of the NAO index.

Figure 1.a shows a manifest peak in the yearly landings and catches per unit effort (CPUE) during 2001 and 2002. Variations in landing and CPUE are not originated by changes in the effort since fluctuations in the gross registered tonnage of purse seiners are small (Figure S2) and the number of vessels targeting anchovy shows a smooth decreasing tendency along these years [24]. Despite the March/April closure of the fishery, monthly landings for the peaking years (insert in Figure 1.a) identify a strong recruitment in 2001 that was able to sustain high catches throughout the winter until February of 2002. Figure 1.a shows that the Julian calendar creates an artifact in the analysis of recruitment data. The high landings of 2002 are the consequence of a successful recruitment in the spawning season of 2001, not in the year 2002 (insert in Figure 1.a). Monthly landings were then aggregated from August to the next July (Figure 1.b), accumulating the production after each spawning season. This representation indicates the very anomalous recruitment of the year 2001 that resulted in landings more than twice the size of the second maxima in the series. Figure 1.b also shows the sensitivity of the stock to environmental conditions: high recruitment occurs during years of negative phase in the North Atlantic Oscillation (NAO) but the NAO alone is not enough to fully explain the recruitment fluctuations. Thus, the main peak of 2001 is not associated with the lowest NAO and the negative NAO in 2006, 2009 or 2010 does not result in high landings.

**Figure 2:**
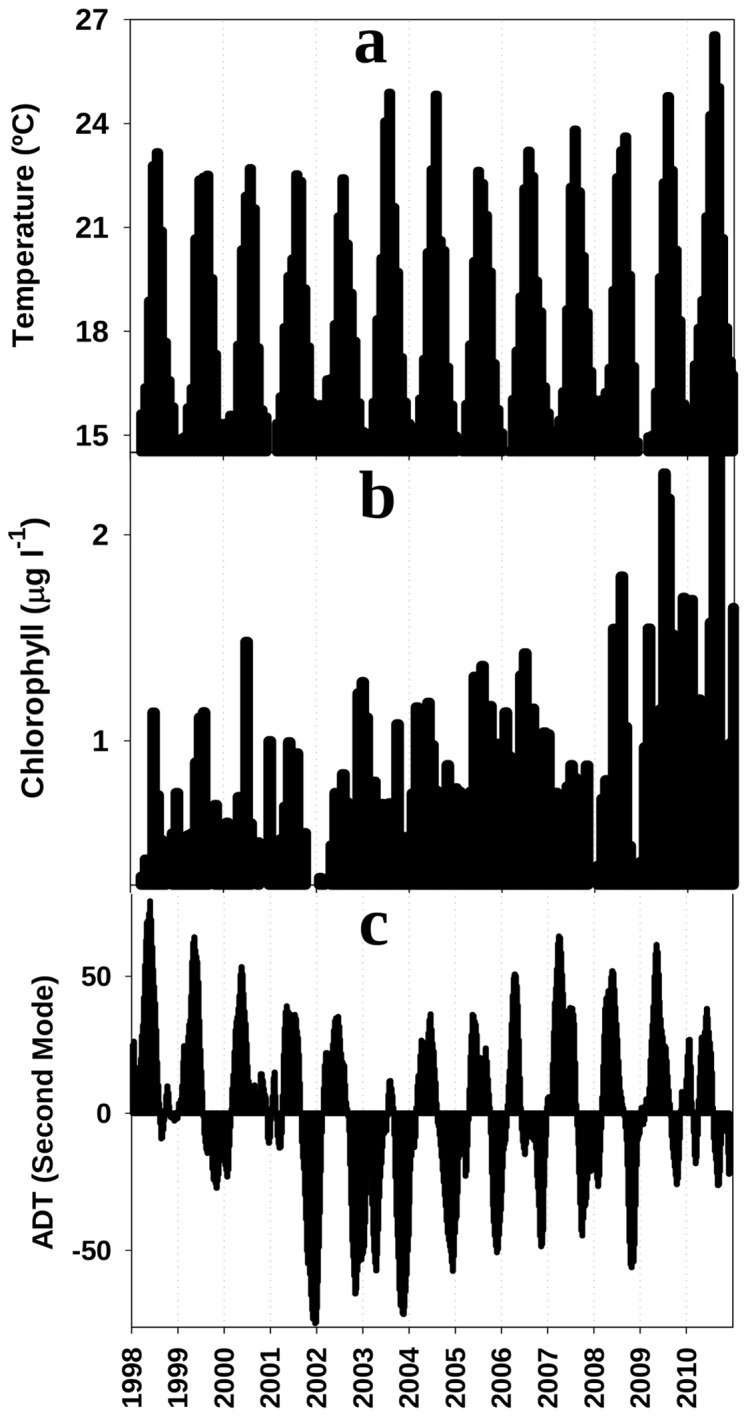
Time series of SST, chlorophyll and ADT mode. Monthly averages of (a) SST and (b) chlorophyll concentration in the northwestern shelf of the Alboran Sea. b) Temporal evolution of the expansion coefficients of the ADT second mode after a coupled singular value decomposition between ADT and CHL fields. Data of this mode were obtained from [17].

The potential connection of this high-recruitment signal with enhanced primary production or a thermal change in the basin is explored in Figure 2. The figure shows the primary production of the shelf dynamical region [32], where anchovy nurse and recruit [33]. Figure 2.a shows the monthly average of sea surface temperature (SST) in the same region as derived from the Pathfinder data. No remarkable feature seems evident in the thermal signal of 2001. Figure 2.b shows the concentration of chlorophyll for the same area as derived from the GlobColour project. The high recruitment of 2001 did not coincide with sustained high concentrations of chlorophyll throughout that year. Instead, chlorophyll fell abruptly in autumn toward the persistent minima of the series.

Chlorophyll in the western Alboran Sea is highly sensitive to the circulation of the AJ-WAG system [32]. Singular value decomposition (SVD) of the absolute dynamic topography (ADT) and sea surface chlorophyll (CHL) fields synthesizes this sensitivity in the expansion coefficient of the ADT second mode [17]. Figure 2.c shows the temporal evolution of this mode. This mode is also atypical in 2001, with an abrupt downward shift after spring and summer toward the minima of the series. Low values of the expansion coefficients of this mode imply a weakening of the circulation system [17].

**Figure 3:**
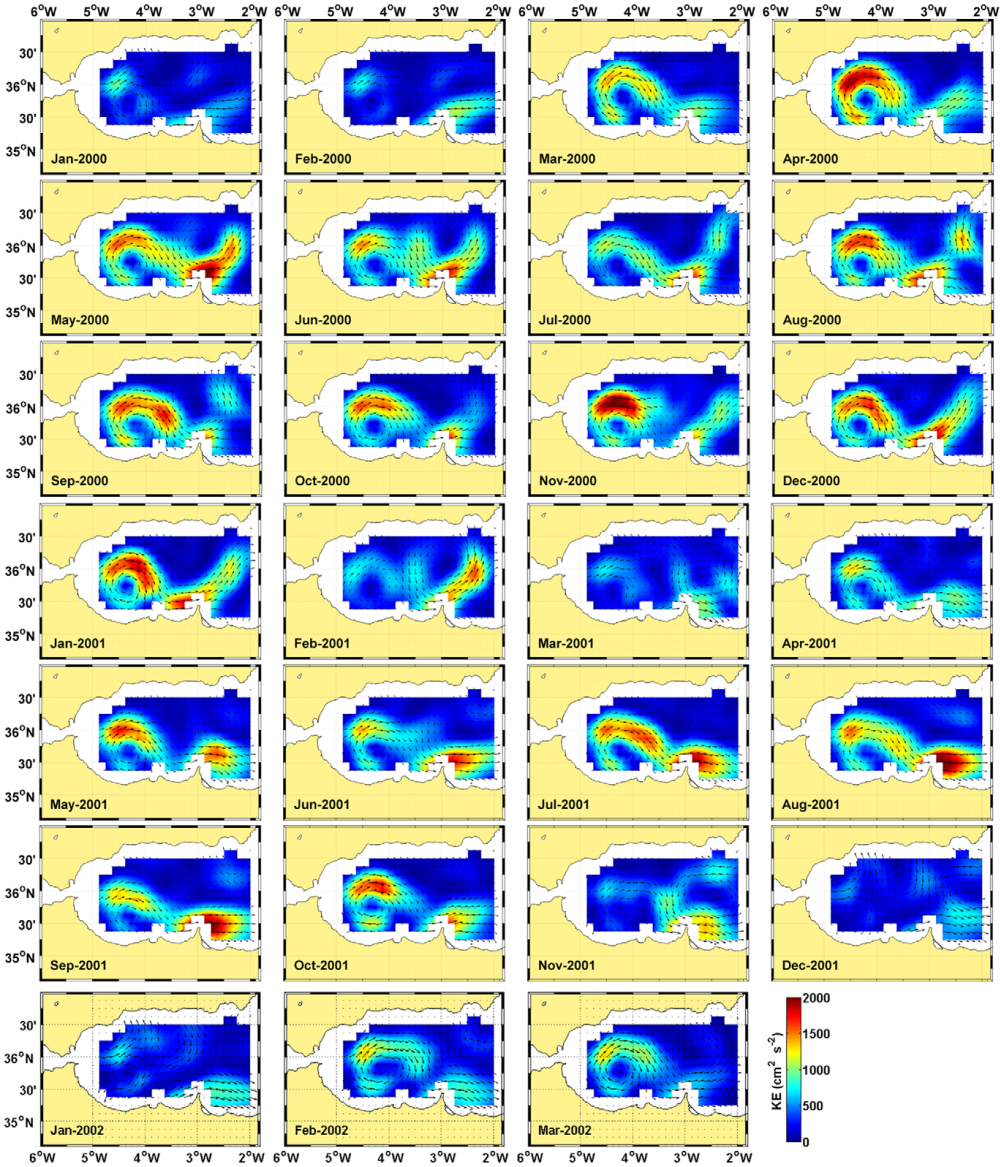
Circulation in the Alboran Sea. Monthly composites of geostrophic circulation and kinetic energy derived from altimetry data.

**Figure 4:**
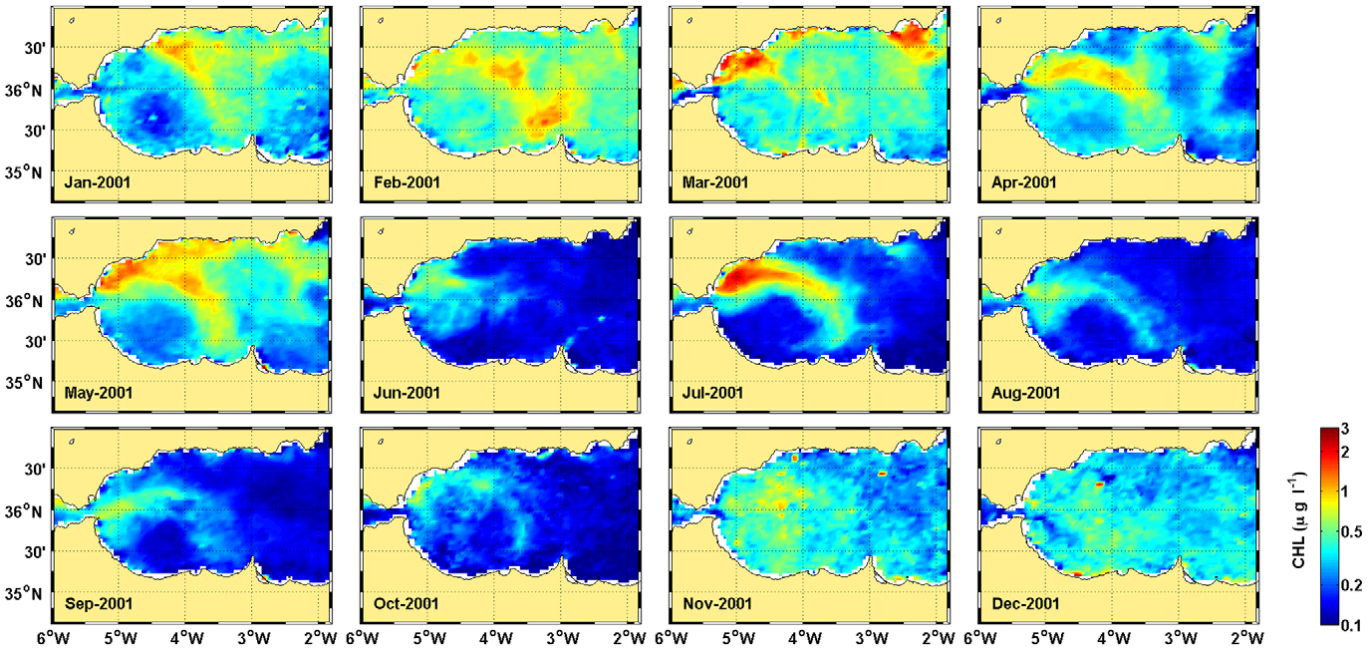
Monthly composites of chlorophyll concentration in 2001.

Figure 3 explores this weakening through monthly composites of the geostrophic circulation and kinetic energy in the Alboran Sea during the period of these abrupt changes. As a contrast with a more standard situation, Figure 3 also shows composite images of the year 2000. From March 2000 to February 2001, the circulation stabilizes in the typical pattern of the basin. The AJ encircles the WAG and then meanders northeastward after reaching Cape Tres Forcas in the African coast (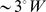) to round the eastern anticyclonic gyre (only partially seen in the figures), cyclonic circulation is observed between both of the gyres. From March 2001 to January 2002, the circulation is very different. During this period, the AJ does not meander northward after Cape Tres Forcas but remains flowing eastward close to the African coast. The energy of the system decreases, particularly after October 2001, when the AJ is not strong enough to activate stable structures in the basin circulation. The consequences for the chlorophyll distribution of these circulation features during 2001 are explored in Figure 4 (Figures S3 S4, S5, S6, S7, S8, S9, S10, S11, S12 provide the full series of chlorophyll and SST from 1998 to 2011). Chlorophyll remains high during spring and early summer, when the AJ is visible as an elongated zone of high chlorophyll. This feature diminishes after July and disappears after October.

**Figure 5:**
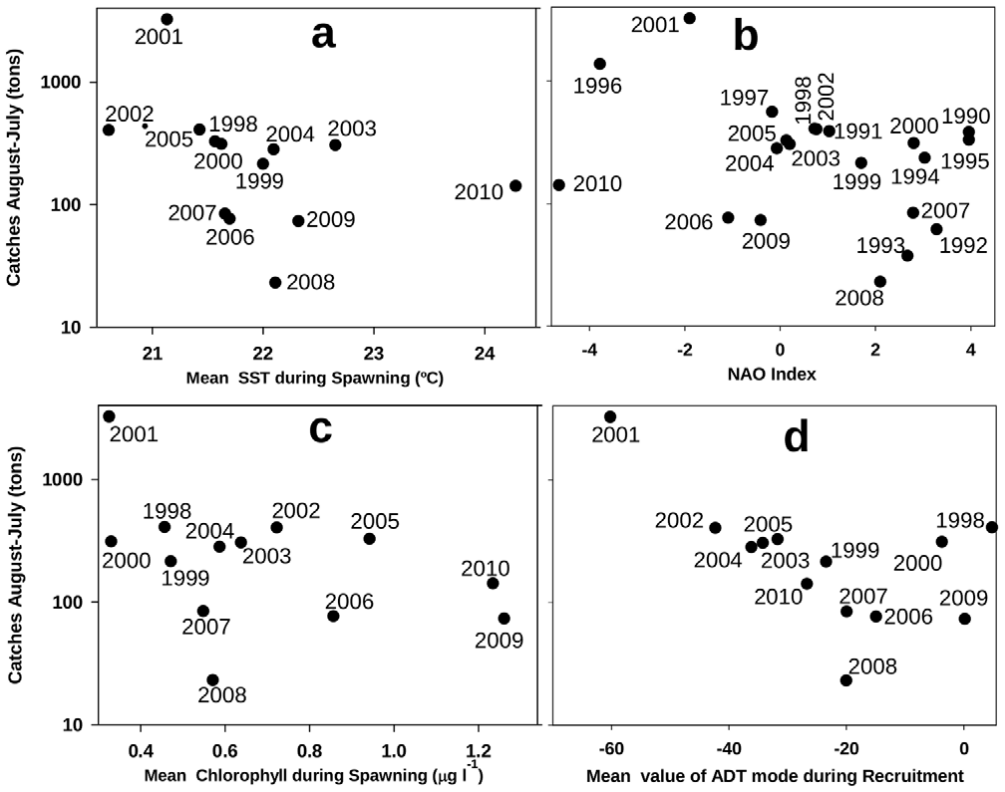
Recruitment versus SST, NAO, chlorophyll concentration and ADT mode. The figure shows the landing of Fig. 1.b versus the mean of (a) SST and (c) chlorophyll concentration at the shelf during spawning period (June to September) as well as (b) the NAO index and (d) the expansion coefficient of the ADT second mode during recruitment (October to December).

There is no evident connection between proxies for abundance (CPUE) and chlorophyll at the time of spawning (Figure 5.c; 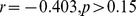), when early stages demand high food concentrations. Despite the control that temperature may exert on the survival of fish larvae [34], the correlation between SST during the spawning season and subsequent catches is also low (Figure 5.a; 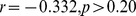). Large-scale environmental indexes, such as NAO, also exhibit poor explanatory power (Figure 5.b; 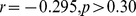), and only ADT at the time of recruitment seems to be connected with catches (Figure 5.d; 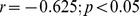). Moreover, a time-lag analysis of recruitment shows that this (0-lag) correlation between ADT and recruitment is the only significant correlation in the series.

In addition to this correlational analysis, a test for one outlier based on a chi-squared distribution of squared differences between the data and sample means [35] was applied to the series of recruitment, SST, chlorophyll and ADT. As in Figure 5, the analysis was implemented for the period between 1998 and 2010, when information is available for all of the environmental variables. The test does not identify as an outlier the chlorophyll in the spawning period of 2001. Additionally, the test does not identify as an outlier the SST in 2001 either at the period of spawning or during the recruitment period. In contrast, the ADT and recruitment during 2001 are the unique outliers of their respective series, with 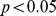 and 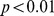, respectively. The conditional distribution for the number of coincidences, 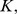 of two outliers in two independent discretized paths of length 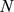 is described by the following hypergeometric distribution [36]:
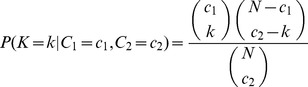
(1)where 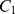 and 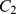 are the number of outliers in each series. For the analysis presented here, 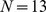, 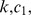 and 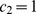 because the chi-squared test detected only one outlier for each of the ADT and recruitment series. This combination results in a low probability (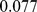) of ADT and recruitment outliers to randomly coincide in the same year of the series.

## Discussion

The year 2001 stands out in the time series analyzed as a strong outlier in both circulation and anchovy recruitment. No coherent signal is observed in other small-pelagic fisheries like sardine and mackerel (Figure S13) or round sardinella [37] what might have suggested an interspecific-interaction origin for the anchovy peak. The landings of these species in 2001 and 2002 are around the series average and there is no evidence of any particular direct, inverse or lagged relationship between anchovy and sardine, mackerel or round sardinella populations at the short term. Together with the correlations emerging from Figure 5, this pattern is an improbable random coincidence that provides evidence to ground the extreme recruitment in that year on the exceptional environment forced by circulation. Indeed, the recruitment of short-lived pelagic species, such as anchovy, is very sensitive to hydrodynamics because of the hydrodynamic control over the trophic environment at spawning areas [38], [39]. Hydrodynamics also have the potential to advect early life stages with reduced motility away from environments that are suitable for recruitment. Marine areas that are optimal for the successful recruitment of clupeoids frequently have relatively high primary production but low advection and turbulence [40], [41].

These conditions do not easily occur together in the Alboran Sea. This basin is productive but as a result of the very intense dynamism forced by the AJ. Thus, the fertilization necessary to produce food for early stages is accompanied by strong currents that are able to advect these stages away from the spawning place. The intensity of this egg and larval transport is well known. There are reported cases of advection within the AJ of fish larvae from the north coast toward deep waters south in the basin [42]. Three-dimensional displacements of anchovy larvae in the intense mesoscale circulation forced by the AJ have also been reported [30]. Larvae removal from the shelf is only activated during northward excursions of the AJ, whereas a jet flowing away from the north shelf contain no anchovy larvae [30]. Indeed, the capacity of a northward-oriented AJ to remove anchovy larvae from the Iberian shelf is very strong. Events of neritic fish-larvae being massively swept away from the northwestern shelf by the AJ are frequently recorded [43]. During these events, most of the shelf is occupied by oceanic larvae that are advected from the open ocean within the AJ.

We postulate that these reported cases and the results presented here reveal the tight control that the AJ exerts over the anchovy recruitment in the Alboran Sea. The field studies of ichthyoplankton in the area demonstrate beyond question the capacity of a strong AJ to sweep neritic larvae from the shelf. This capacity depends on the strength of the AJ-WAG system, as diagnosed in Figures 2, 3 and 5. Its operation over the years analyzed here, 1998 to 2010, connects the signals of AJ-WAG strength/weakness (Figures 2.b and 3) and recruitment failure/success (Figure 1.b) during this period. The AJ-WAG system is too energetic during standard years to favor a successful recruitment. The intense AJ-WAG system forces surface fertilization and high chlorophyll concentration during typical years (Figure 2). However, the positive influence of this fertilization on recruitment is strongly counterbalanced by the advection resulting from energetic currents. Recruitment might be dampened due to spatial constraints imposed by the AJ on the spawning and nursery grounds, most likely affecting the amenable area rather than the available food. Other environmental factors, such as temperature, seem to play a minor role in the process (Figure 5.a–b). The weakness of the AJ-WAG system during the year 2001 creates an exceptional setting over this background scenario of high productivity and high advection. The unusual circulation releases most of the northern Alboran Sea from the intense currents of the eastern Alboran gyre, the cyclonic circulation between anticyclones and even of the WAG after October (Figure 3). During late spring and summer, the AJ-WAG is weak but is not totally shut down (April to October 2001 in Figure 3) and still allows high chlorophyll concentrations (Figures 2 and 4). After summer, the AJ-WAG system weakens to such an extent that it disappears from the basin, and the amount of energy is too low to firmly activate fertilization mechanisms. This cessation of the AJ-WAG system during three months (from November 2001 to January 2002) is exceptional in the series of altimetry images (Figures S14, S15, S16, S17, S18). The exceptionality of this period in the circulation of the Alboran Sea is also confirmed by the time series of basin currents as reconstructed through modeling [44]. Under these conditions, the production of the shelf persistently decreases toward the lowest values of the series (Figure 2). The lack of fertilization at the northwestern shelf is also evident from field studies in this period [45]. Therefore, there seem to be two phases of the AJ-WAG system in 2001: a first period of anomalous weakness during spawning in spring/summer and a full collapse during recruitment in autumn/winter.

The amount of production during spawning cannot explain the successful recruitment of 2001 because the mean chlorophyll of that year is the lowest of the series (Figure 5). The phytoplankton data suggest a profound trophic shift at the northern shelf of the Alboran Sea during 2001 and early 2002, as revealed by the substitution of the diatom-dominated community by a coccolithophorid and dinoflagellate community [45]. This shift agrees with a less dynamic environment in which diatom-based communities are not favored [46]. Further, dinoflagellates and protists have been found in other areas at greater proportions than diatoms in the gut of young anchovy [47], [48], thereby reinforcing the evidence that the food environment was not adverse for anchovy larvae during the spring and summer of 2001. This situation is also supported by estimates of the daily growth (based on otoliths) of anchovy larvae in the northern Alboran Sea, with enhanced growth in the 2001 spawning season [27]. Consequently, the weakening of the AJ-WAG system favors retention in the shelf but, despite the concomitant decrease of primary production, apparently had no negative effect on the food available to anchovy larvae and postlarvae during early 2001.

However, the trophic environment becomes very adverse in autumn/winter because primary production collapses with the collapse of the AJ-WAG system (Figure 2). Post-larval stages stay near the coast, but juveniles increasingly spread over the shelf as the season progresses, and a larger size provides these juveniles more motility [49]. As their motility drives their behavior away from the planktonic realm, this change decreases the sensitivity to food concentration relative to that of earlier stages [50]. If physical forcing is relaxed, post-larval spread over stable areas may favor faster than usual growth even if production is reduced [21]. Several authors propose that small pelagic species use “continual testing” of the environment to take advantage of profitable spatial configurations that contribute to population resilience [51]. Indeed, during 2001, the northwestern shelf of the Alboran Sea had very low fertilization after summer, the period when the juvenile stages explore the shelf (Figures 3 and 4 [45]). Apparently, this lack of fertilization had no negative impact on the recruitment (Figure 1.b). The juvenile season of 2001 is the only period in the series when the AJ-WAG system persistently collapsed (Figures 2.b and 3). The consequences of this collapse on the survival of juveniles should be explored in the context of juvenile vs. larval stages as modulators of recruitment. The role of late larvae/early juveniles in shaping class 0 strength has received little attention but has supporting evidence and is frequently interpreted in terms of density-dependent mechanisms [52]. The velocities involved in a well-developed AJ-WAG system are too high (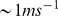) for the system to be a suitable habitat for these stages. Thus, a severe weakening or cessation of the AJ-WAG system not only prevents the offshore transport of early stages with limited motility but also widens the portion of the shelf without energetic currents, thus providing a reduced density-dependent competition by increasing the amount of calm areas that are available to early stages. Anchovy largely constrains its recruitment to the northwestern shelf of the Alboran Sea what makes the stock extremely sensitive to the fluctuations of the local environment there, as described above. Other small pelagics with a broader distribution, like sardine, seem to be less sensitive to the local regime generated by the AJ as it enters the Mediterranean (Figure S13).

In summary, we contend here that anchovy recruitment in the region is inhibited by the advection and dispersion of larvae and post-larvae during periods of strong advection by the AJ. Food availability is not the primary limiting factor in the region. During standard years, the intense currents fertilize the region and sustain this anchovy stock but also keep recruitment very low. Exceptional years involve a weakening, but not a total collapse, of the AJ-WAG during spring and summer. This weakening provides adequate food and stability to larvae and postlarvae. The total collapse of the AJ-WAG after summer seems to have a positive effect on recruitment. These exceptional years open an optimal window (*sensu* Cury and Roy [40]) for a recruitment that is quasi-permanently kept at the edge by the AJ to shift to the right of the dome-shaped curve of [40] hypothesis. However, this shift involves such an improbable combination of events in the Alboran Sea that this window can only be qualified as extremely narrow in time and very sensitive to the circulation in the Strait of Gibraltar. This circulation is in a transient state due to modifications in the thermal and hydric balances of the Mediterranean [53], a scenario that will have consequences, though not necessarily negative, on future anchovy landings in the western Mediterranean.

## Materials and Methods

Environmental control of the recruitment of small pelagic fishes is frequently analyzed in the context of meteorological records. This approach implicitly assumes that atmosphere-sea interaction somehow reflects the meteorological information into ocean circulation and biological production. Sea surface topography derived from satellite altimetry is more directly connected to ocean dynamics as a tracer of synoptic and high-resolution geostrophic circulation [54]. However, this information is less frequently explored despite now being available to the scientific community as accessible products and maps. This section describes the sources and nature of altimetry information in the context of its explanatory power for proxies for anchovy abundance in the Alboran Sea. This section also describes the sources of fishery records in the Alboran Sea as well as the origin of sea surface temperature and color data. The length of the time series is shorter for environmental (13 years) than for fishery (23 years) or NAO index data because the operation of the color sensor began in late 1997. Although the long set of catch data is used in the manuscript to show time tendencies and their connection with NAO, the joint analysis of recruitment and environment is restricted to the eleven years when a consistent set of data is available for both the fishery and environment.

### Fisheries data

The anchovy recruitment in the North Alboran Sea is strongly dependent on the 0 age-class, and both landings and CPUE are frequently used as abundance proxies in the area ( [24] and references therein). Several data sources have been used to compile the time series of anchovy landings and CPUE in the Alboran Sea. In addition, a precise analysis of the historical information for the small pelagic fishery in the basin is also available [55]. This analysis dates back to initial landings data from 1925, with increasing detail for more recent records. The continuation of that work [19], [56] provided exhaustive information, including monthly landing and CPUE data disaggregated by species and port, for the period between 1985 and 1995. The General Fisheries Commission for the Mediterranean, through its annual assessment made by the Working Group on the stock assessment of small pelagic species, provides yearly landing and CPUE data since 1990. Monthly landing (but not CPUE) data are again available since the year 2000 from the website of the Regional Government (IDAPES database; www.juntadeandalucia.es/agriculturaypesca/idapes). Since 1999, the fishery is affected by closures during March and April; thus, the data contain gaps for these months and are excluded from the seasonal analysis.

The yearly catches analyzed in this paper include data after 1986, when this fleet only landed catches from fishing grounds north of 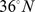 [55]. The port of Malaga registers almost 85% of all of the anchovy landings of the northern Alboran Sea, and after several corrections for anchovy fished elsewhere but disembarked at Malaga port, the data of anchovy landed in the Port of Malaga are routinely used to assess landings in the N. Alboran Sea [57]. The CPUE data from the General Fisheries Commission for the Mediterranean (GFCM) and other reports [19] exactly coincide at the years of overlapping (1990 to 1995), indicating that both are the same series of data. Since the late 1990s, the fishery has been closed between March and April, and the seasonal analyses presented in this paper exclude these years. All of the available assessments of this fishery indicate that recruits (age 0) support the bulk of the fishery [24], and therefore, that the peak of catches in autumn/winter are the result of spawning in the preceding spring/summer [24]. In addition, these assessments also indicate that CPUE is a good index for the relative abundance of spawners in the area [58], what is also evident in the averages of both age in catches (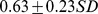) and the percentage of mature individuals at age 0 and 1 (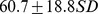 and 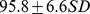 respectively) [24].

### Altimetry data

The ADT data are delayed-time (dt) gridded and merged products with a spatial resolution of 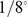 and weekly temporal resolution [59], [60]. These data were provided by AVISO (http://www.aviso.oceanobs.com), covering the entire Mediterranean Sea and combining information from different missions, significantly improving the estimation of mesoscale signals [61], [62].

The KE is calculated as:
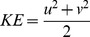
where the velocity components, 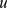 and 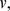 were derived from the geostrophic approximation:



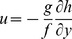


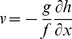
where 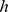 is the ADT, 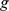 is the gravitational acceleration, and 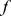 is the Coriolis parameter.

To investigate the combined spatial and temporal covariability between ADT data and CHL, an SVD technique was employed [17]. The SVD was performed on the cross-covariance matrix between the non-normalized values of each field (CHL anomalies and ADT anomalies) to identify pairs of coupled spatial patterns and their temporal variation. The first SVD mode is associated to changes in the sea level of the whole basin in response to variations of atmospheric pressure, whereas the second mode synthesizes the dynamics of circulation structures that are able to control anchovy recruitment in the Alboran Sea [17].

### Sea Surface Temperature data

The SST data used in this study correspond to AVHRR Ocean Pathfinder SST and MODIS L3 SST 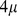. The AVHRR Ocean Pathfinder SST data were obtained from the Physical Oceanography Distributed Active Archive Center at the NASA Jet Propulsion Laboratory, (http://podaac.jpl.nasa.gov/). We used the 4 km Pathfinder version 5 SST Project, which is a new reanalysis of the earlier AVHRR version of the Pathfinder data set that has been distributed since the early 1990s [63]. We used the monthly nighttime data and the period was comprised between 1998 and 2009. The MODIS L3 SST 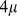 were obtained from the Oceancolor website (http://oceancolor.gsfc.nasa.gov). We used the monthly nighttime data with 4 km of spatial resolution for years comprised between 2010 and 2011.

### Sea Surface Color data

Sea surface chlorophyll a data were downloaded from the GlobColour Project (http://www.globcolour.info/). This source produces global ocean color maps (Level-3) by merging data since 1998 from the three sensors SeaWiFS, MODIS and MERIS. Surface chlorophyll a data correspond to a product of chlorophyll a case I water based on the GSM merging method [64], [65]. This method provides the best fit to in-situ chlorophyll a concentration and has the added advantages of providing other products and allowing researchers to calculate pixel-by-pixel error bars. With these data sets, the cloud cover is reduced, and therefore, more useful images become available. The spatial and temporal resolutions of these composite images were 4.6 km and monthly, respectively.

### North Atlantic Oscillation index

The winter (December through March) index of the NAO is based on the difference of normalized sea level pressure between Lisbon, Portugal, and Stykkishol- mur/Reykjavik, Iceland, since 1864. The NAO index data were provided by the Climate Analysis Section(NCAR) [Boulder, USA], (http:// www.cgd.ucar.edu/cas/jhurrell/indices.html) [66].

## Supporting Information

Figure S1 Map of the Alboran Sea with standard circulation structures. AJ, WAG and EAG stand respectively for the Atlantic Jet as well as the western and Eastern anticyclonic gyres.
(TIFF)

Figure S2 Evolution of the total GRT of purse seiners in the NW Alboran (CopeMed II, 2011).
(TIFF)

Figure S3Monthly composites of chlorophyll concentration (CHL, in 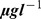) between 1998 and 2000.(TIFF)

Figure S4Monthly composites of chlorophyll concentration (CHL, in 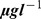) between 2001 and 2003.(TIFF)

Figure S5Monthly composites of chlorophyll concentration (CHL, in 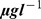) between 2004 and 2006.(TIFF)

Figure S6Monthly composites of chlorophyll concentration (CHL, in 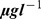) between 2007 and 2009.(TIFF)

Figure S7Monthly composites of chlorophyll concentration (CHL, in 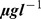) between 2010 and 2011.(TIFF)

Figure S8Monthly composites of Sea Surface Temperature (SST, in 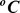) between 1998 and 2000.(TIFF)

Figure S9Monthly composites of Sea Surface Temperature (SST, in 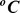) between 2001 and 2003.(TIFF)

Figure S10Monthly composites of Sea Surface Temperature (SST, in 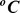) between 2004 and 2006.(TIFF)

Figure S11Monthly composites of Sea Surface Temperature (SST, in 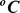) between 2007 and 2009.(TIFF)

Figure S12Monthly composites of Sea Surface Temperature (SST, in 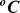) between 2010 and 2011.(TIFF)

Figure S13 Yearly catches of small pelagic fish in the NW Alboran Sea during the last decade (data only reliable since 2000). Source: Andalusian Regional Government IDAPES database (http://www.juntadeandalucia.es/agriculturaypesca/idapes/). Black and white circle are anchovy and sardine respectively while the white square is mackerel.
(TIFF)

Figure S14Monthly composites of geostrophic circulation and kinetic energy (KE, in 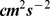) derived from altimetry between 1998 and 2000.(TIFF)

Figure S15Monthly composites of geostrophic circulation and kinetic energy (KE, in 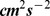) derived from altimetry between 2001 and 2003.(TIFF)

Figure S16Monthly composites of geostrophic circulation and kinetic energy (KE, in 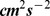) derived from altimetry between 2004 and 2006.(TIFF)

Figure S17Monthly composites of geostrophic circulation and kinetic energy (KE, in 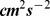) derived from altimetry between 2007 and 2009.(TIFF)

Figure S18Monthly composites of geostrophic circulation and kinetic energy (KE, in 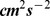) derived from altimetry between 2010 and 2011.(TIFF)
