# Supplementary figures and images for: Recruiting at the Edge: Kinetic Energy Inhibits Anchovy Populations in the Western Mediterranean
Source: PLoS One. 2013 Feb 25;8(2):e55523. doi: 10.1371/journal.pone.0055523 (PMC3581504; doi:10.1371/journal.pone.0055523)

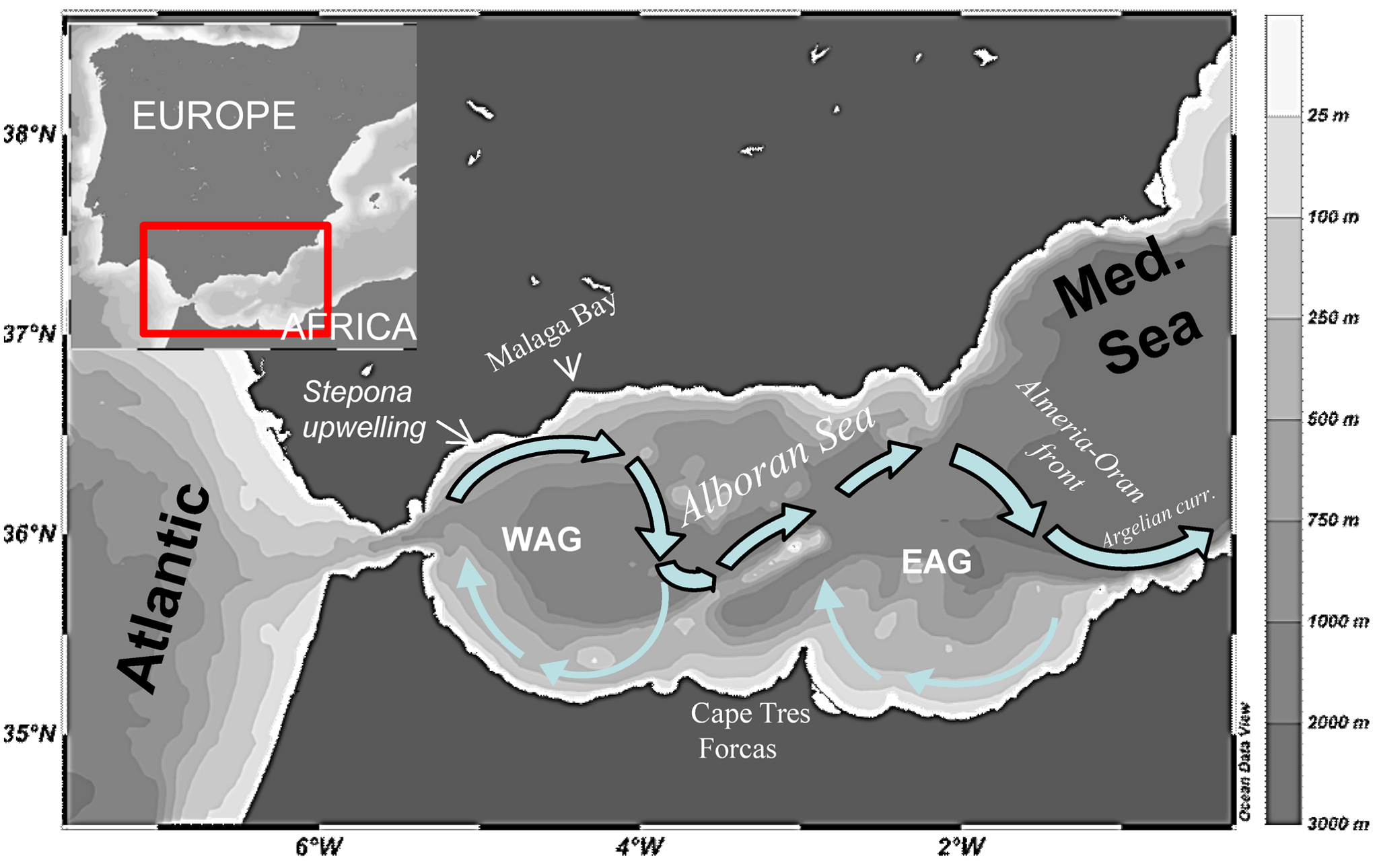

Supplement: Figure S1 — Map of the Alboran Sea with standard circulation structures. AJ, WAG and EAG stand respectively for the Atlantic Jet as well as the western and Eastern anticyclonic gyres. (TIFF) [file pone.0055523.s001.tiff]

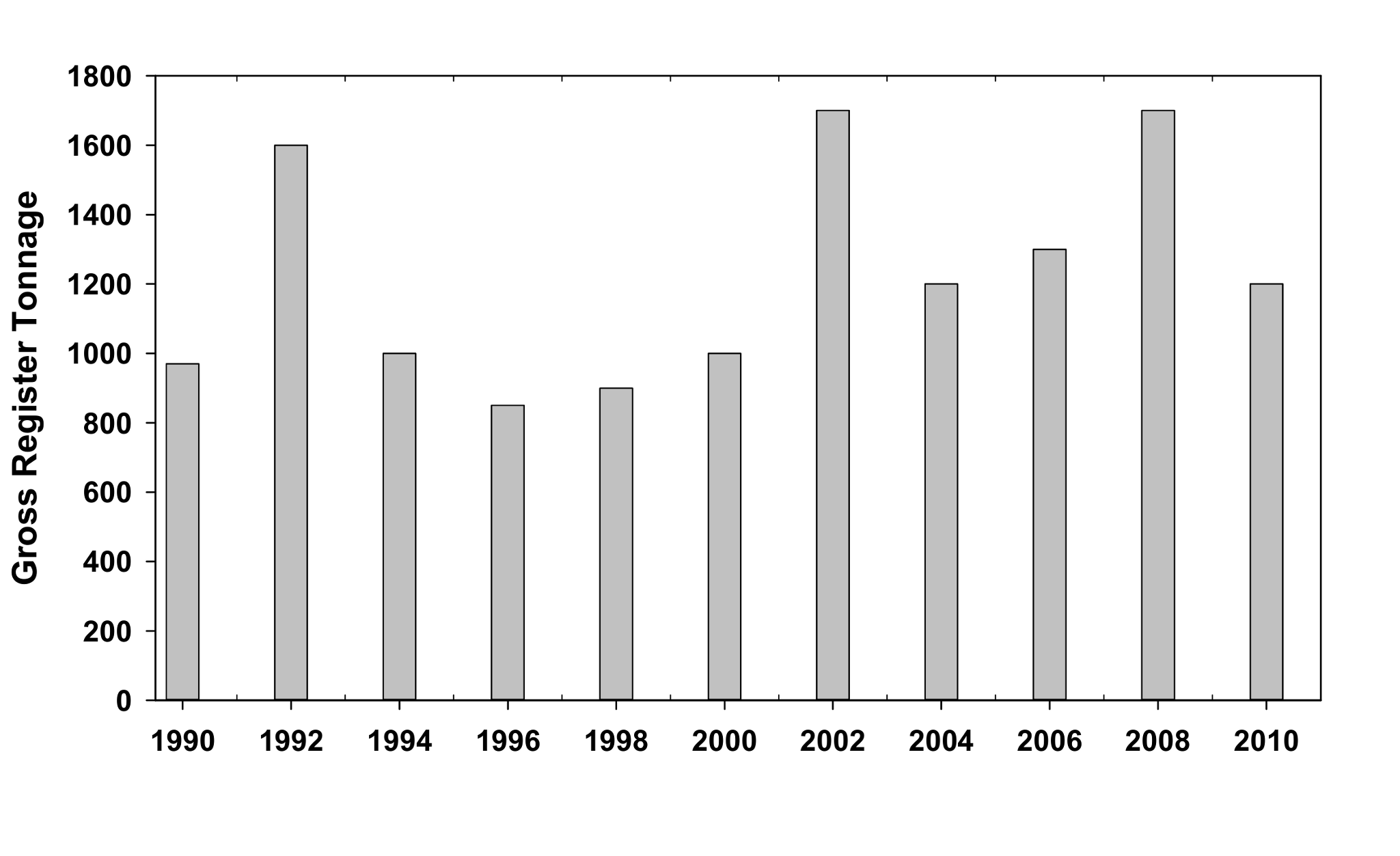

Supplement: Figure S2 — Evolution of the total GRT of purse seiners in the NW Alboran (CopeMed II, 2011). (TIFF) [file pone.0055523.s002.tiff]

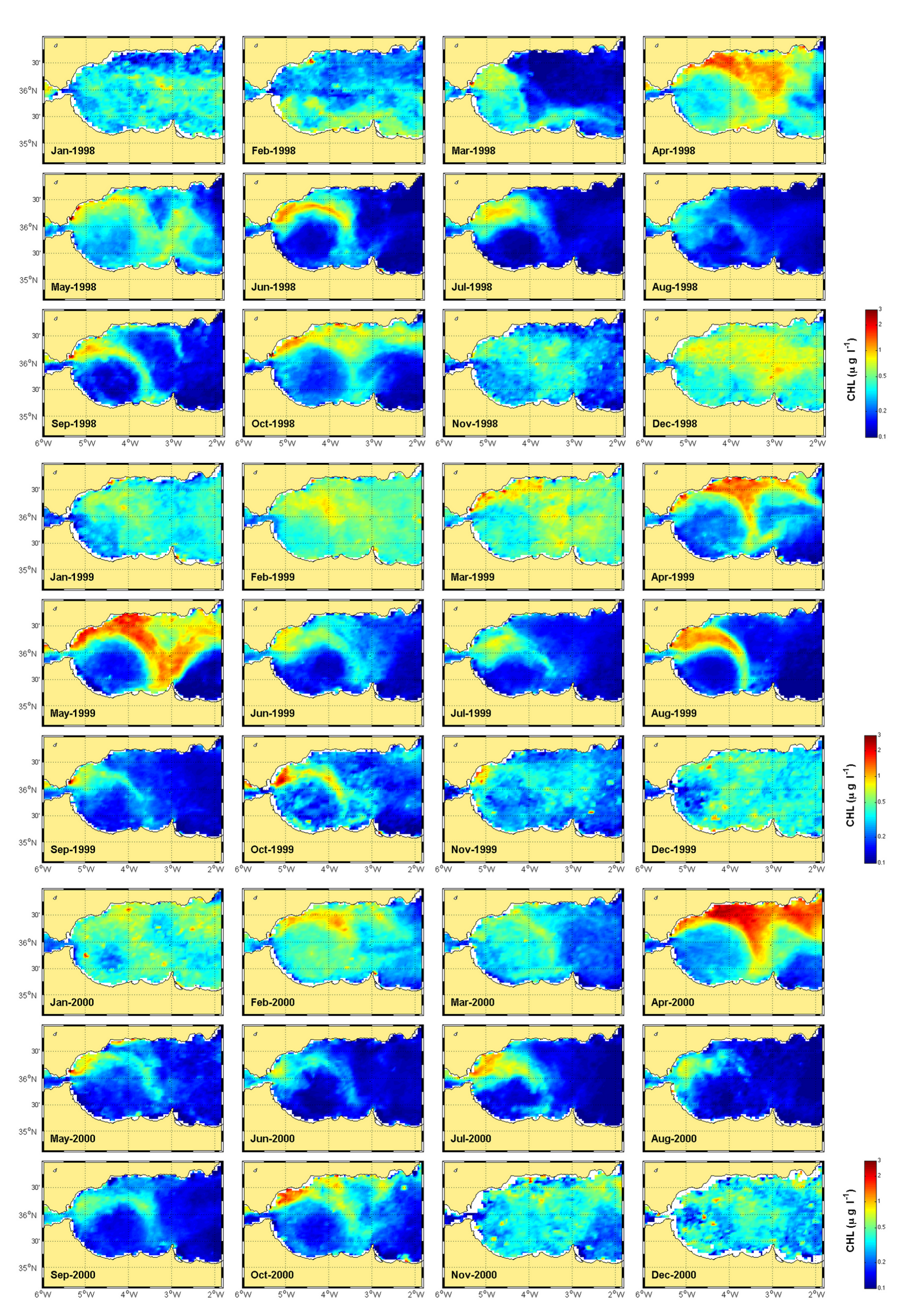

Supplement: Figure S3 — Monthly composites of chlorophyll concentration (CHL, in ) between 1998 and 2000. (TIFF) [file pone.0055523.s003.tiff]

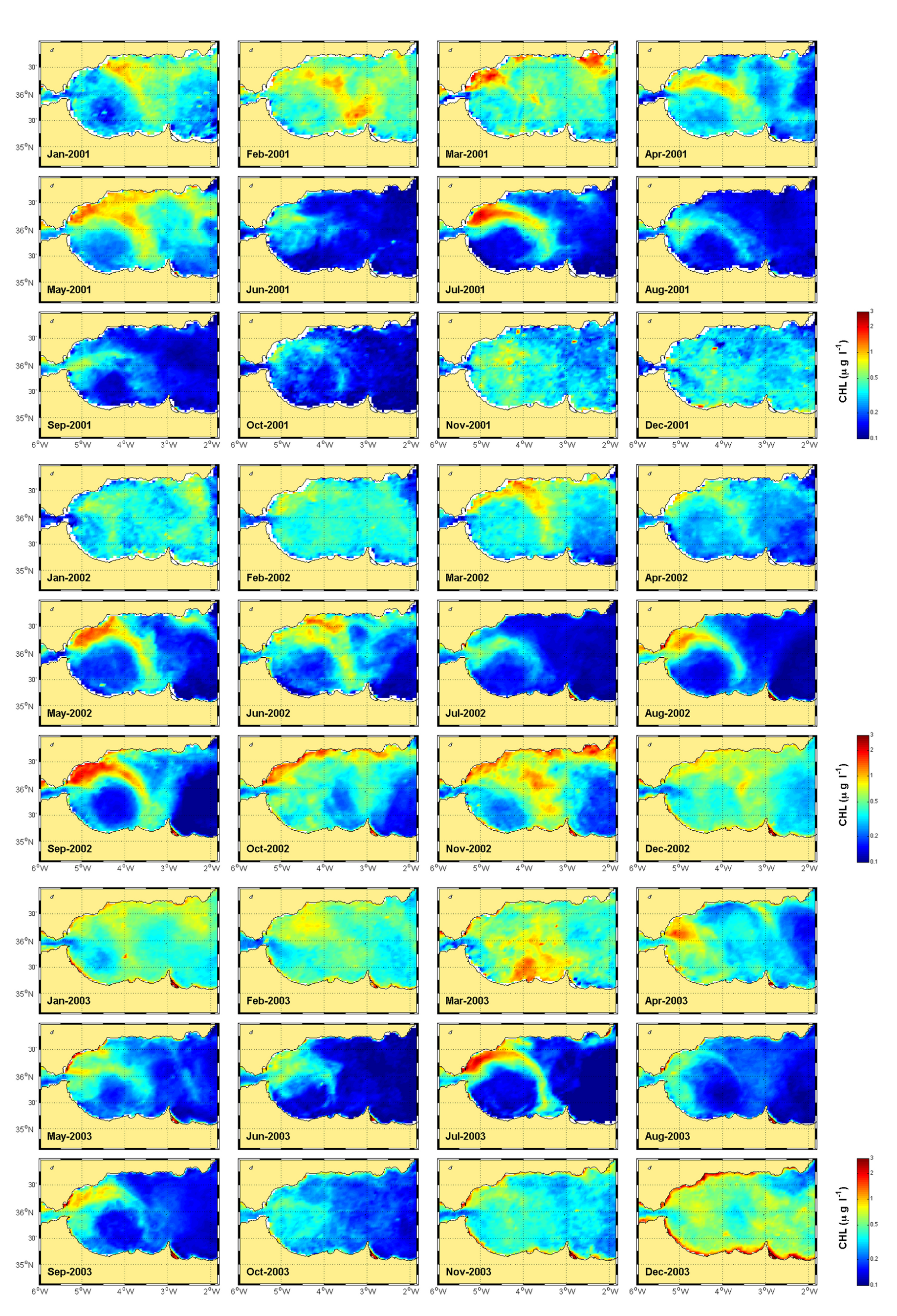

Supplement: Figure S4 — Monthly composites of chlorophyll concentration (CHL, in ) between 2001 and 2003. (TIFF) [file pone.0055523.s004.tiff]

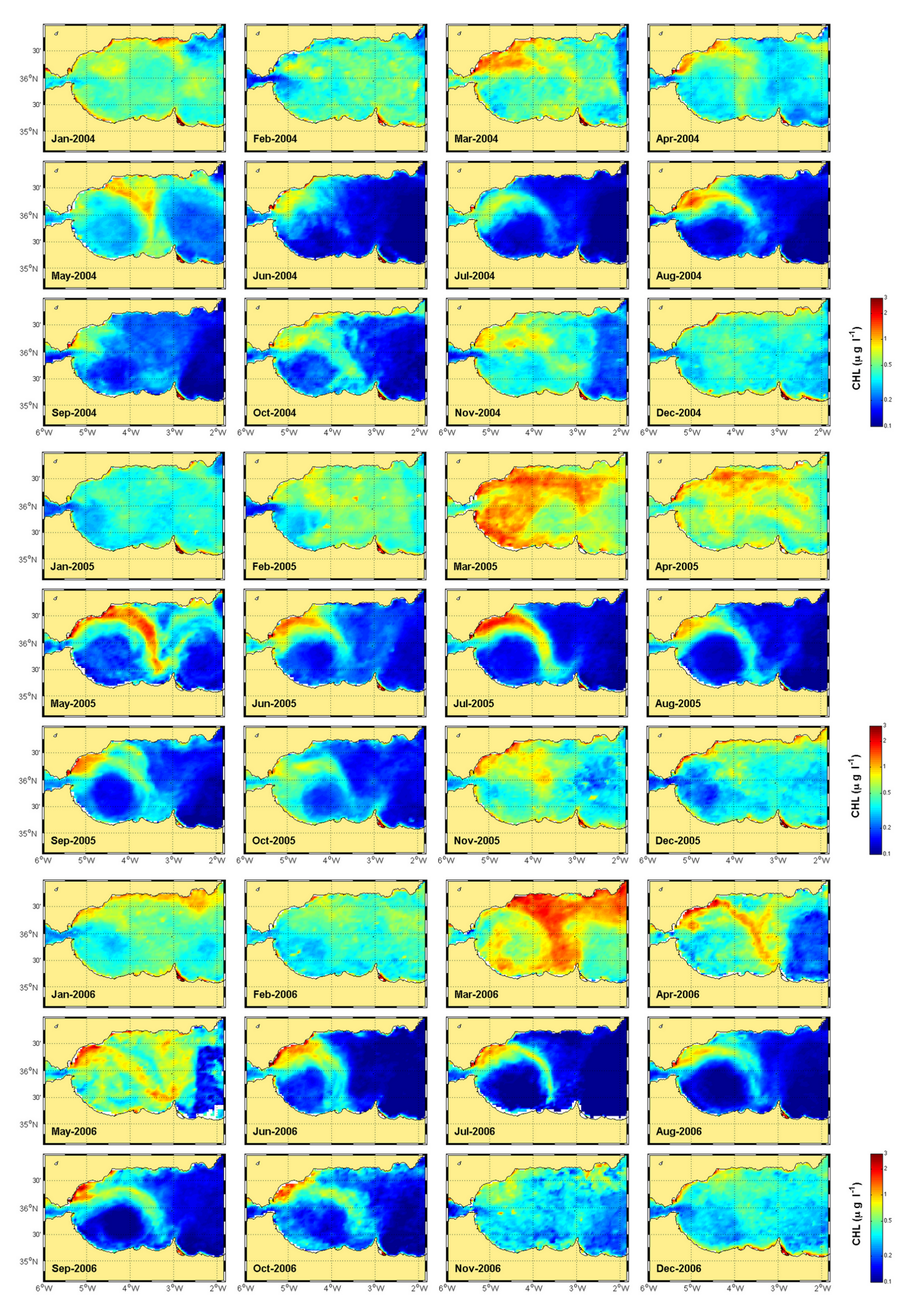

Supplement: Figure S5 — Monthly composites of chlorophyll concentration (CHL, in ) between 2004 and 2006. (TIFF) [file pone.0055523.s005.tiff]

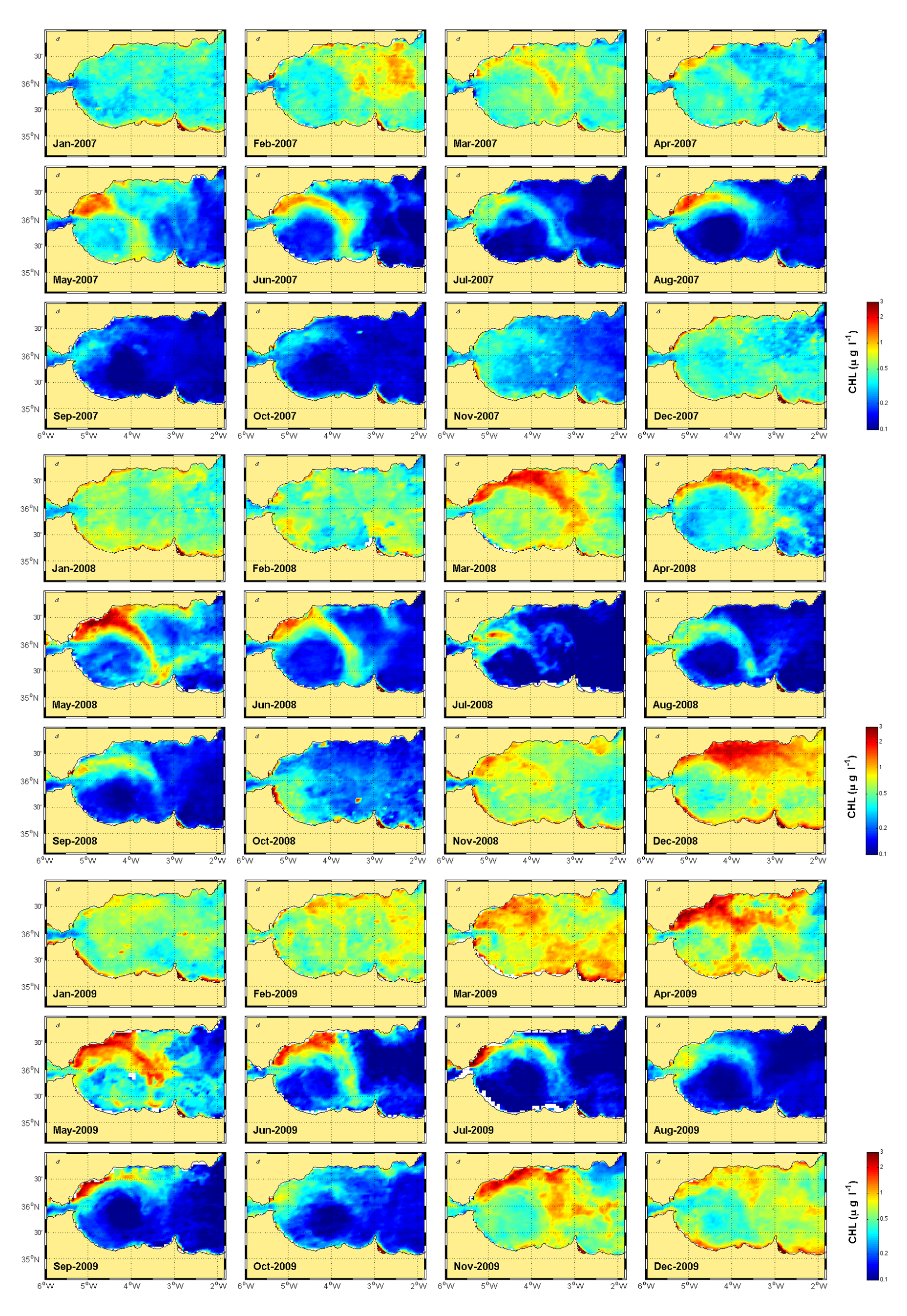

Supplement: Figure S6 — Monthly composites of chlorophyll concentration (CHL, in ) between 2007 and 2009. (TIFF) [file pone.0055523.s006.tiff]

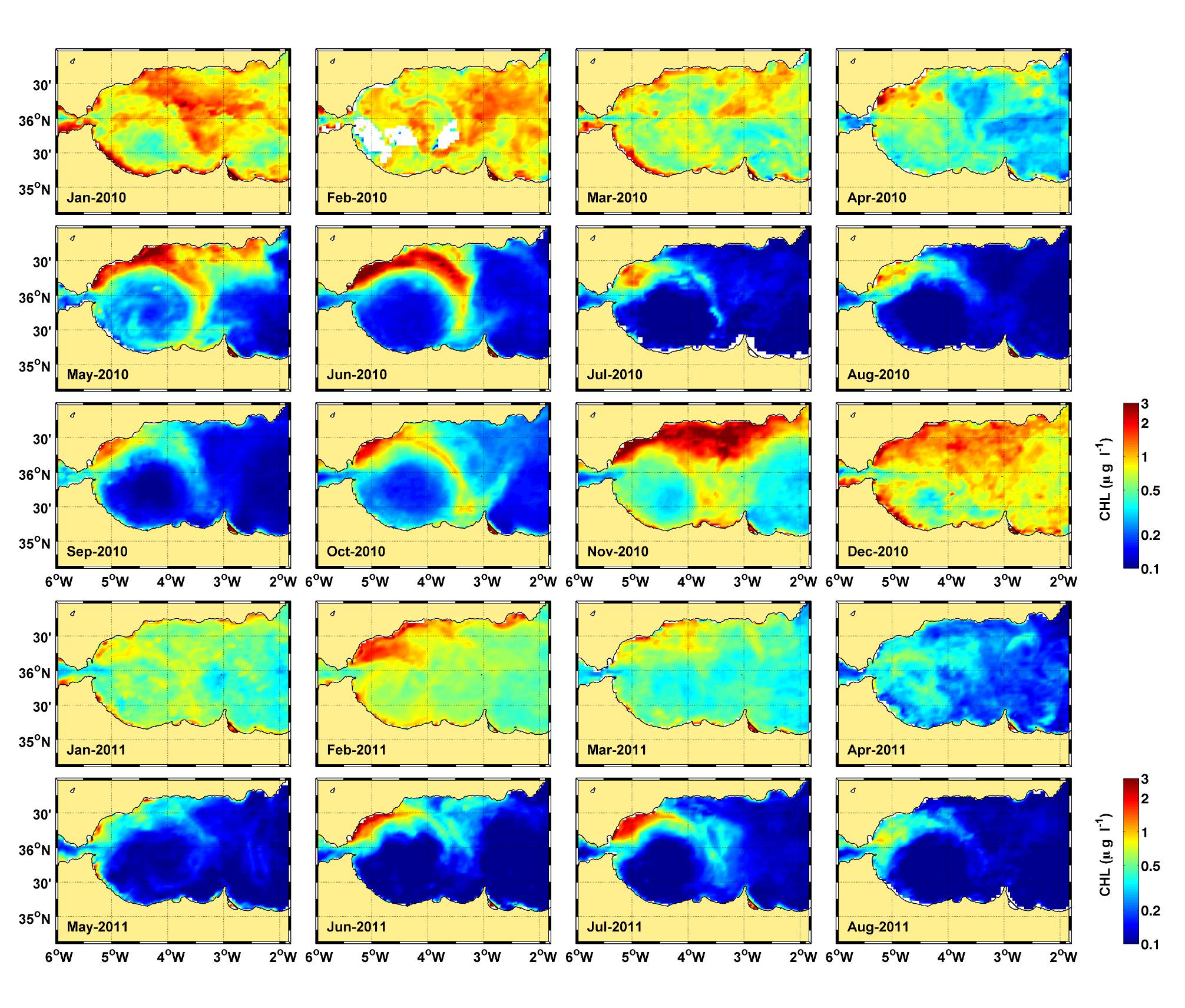

Supplement: Figure S7 — Monthly composites of chlorophyll concentration (CHL, in ) between 2010 and 2011. (TIFF) [file pone.0055523.s007.tiff]

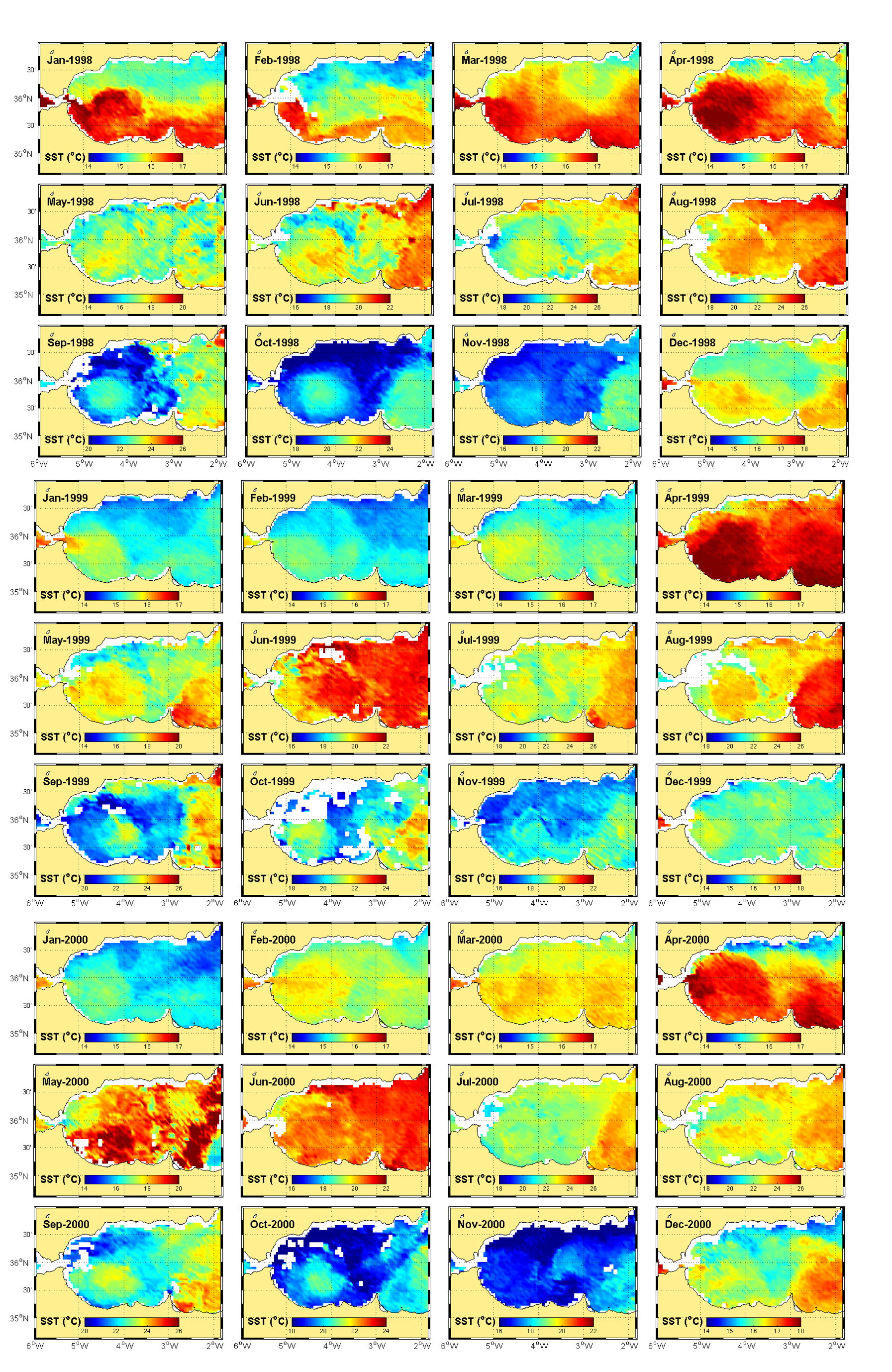

Supplement: Figure S8 — Monthly composites of Sea Surface Temperature (SST, in ) between 1998 and 2000. (TIFF) [file pone.0055523.s008.tiff]

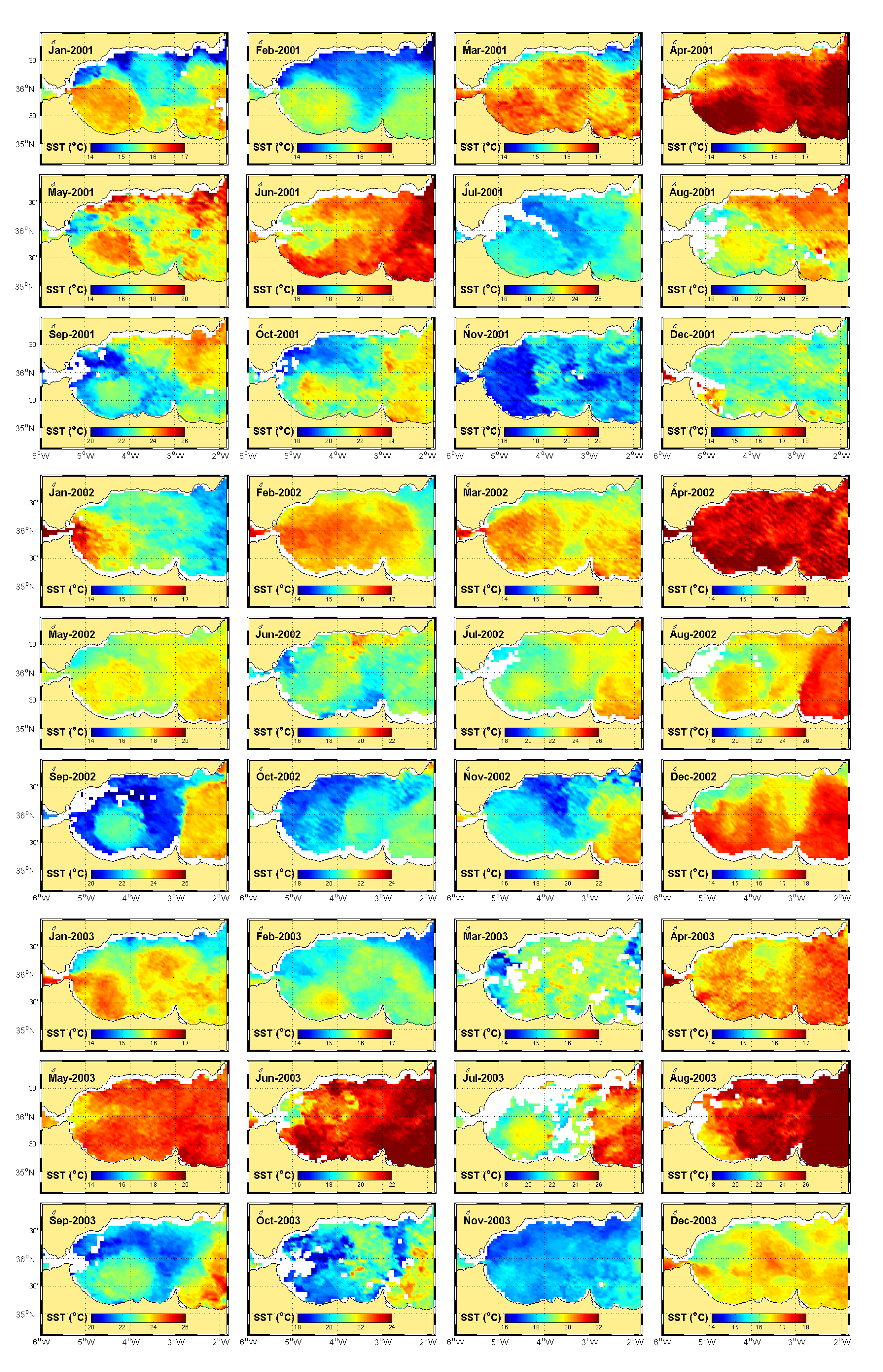

Supplement: Figure S9 — Monthly composites of Sea Surface Temperature (SST, in ) between 2001 and 2003. (TIFF) [file pone.0055523.s009.tiff]

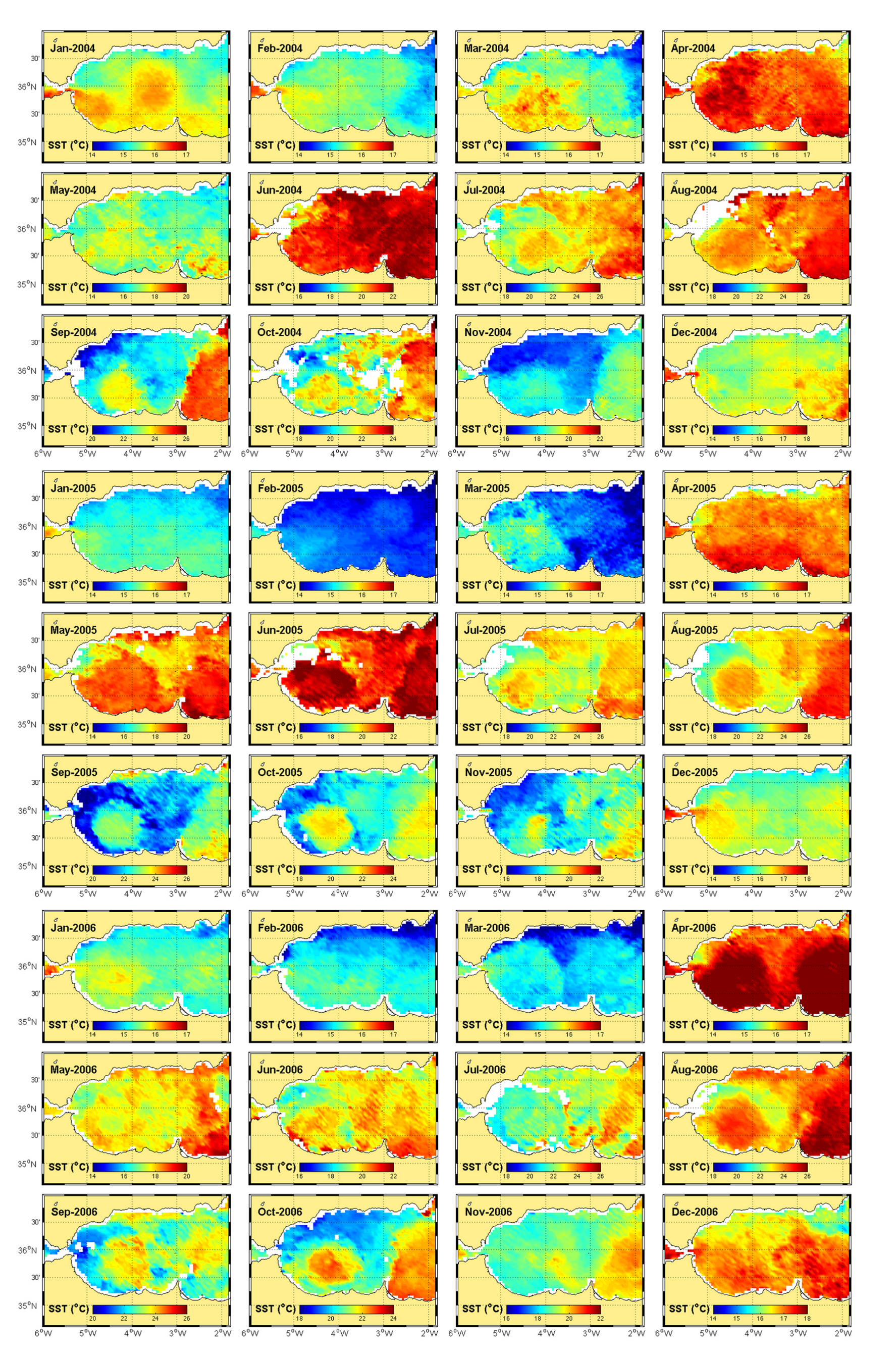

Supplement: Figure S10 — Monthly composites of Sea Surface Temperature (SST, in ) between 2004 and 2006. (TIFF) [file pone.0055523.s010.tiff]

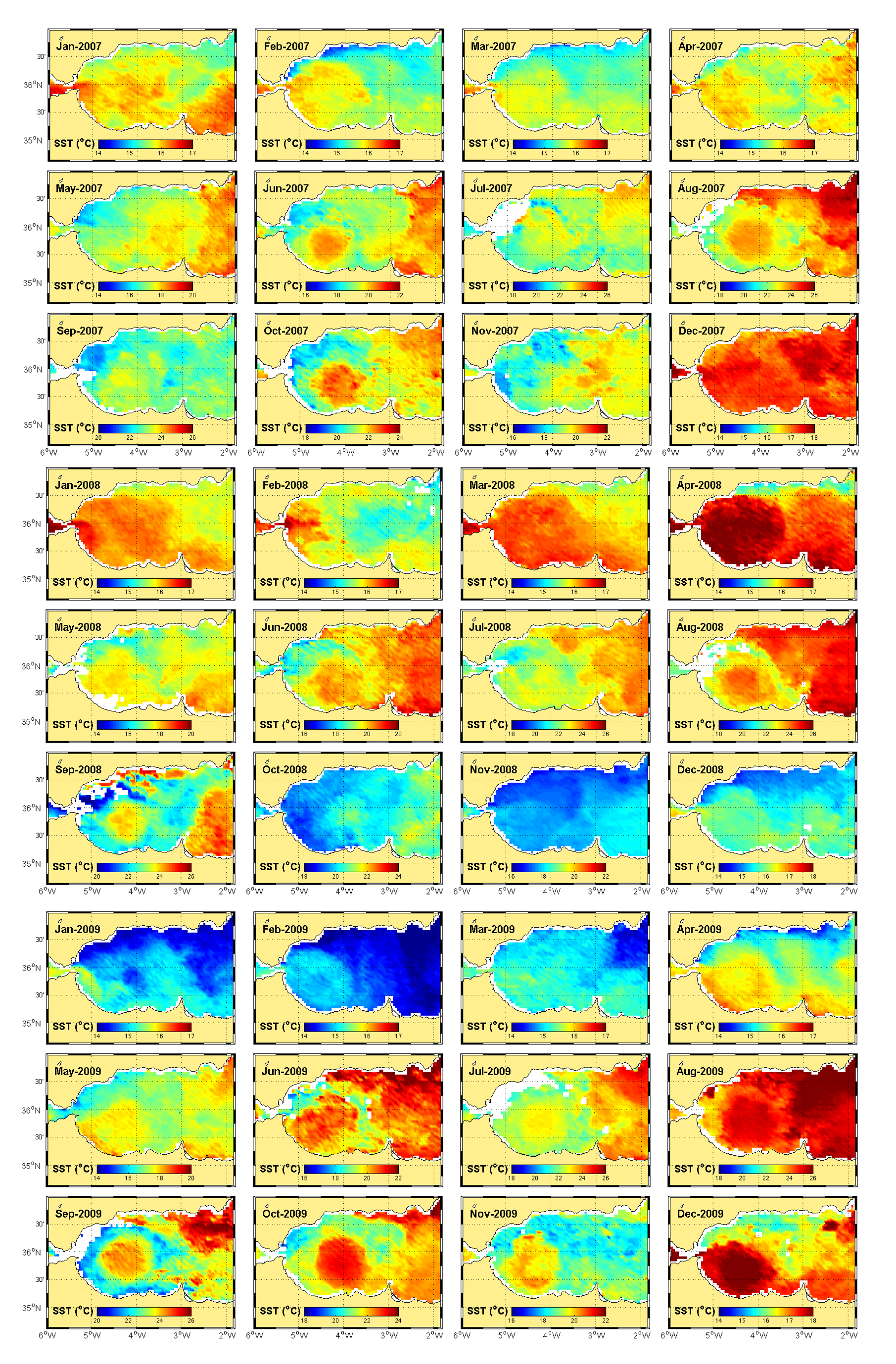

Supplement: Figure S11 — Monthly composites of Sea Surface Temperature (SST, in ) between 2007 and 2009. (TIFF) [file pone.0055523.s011.tiff]

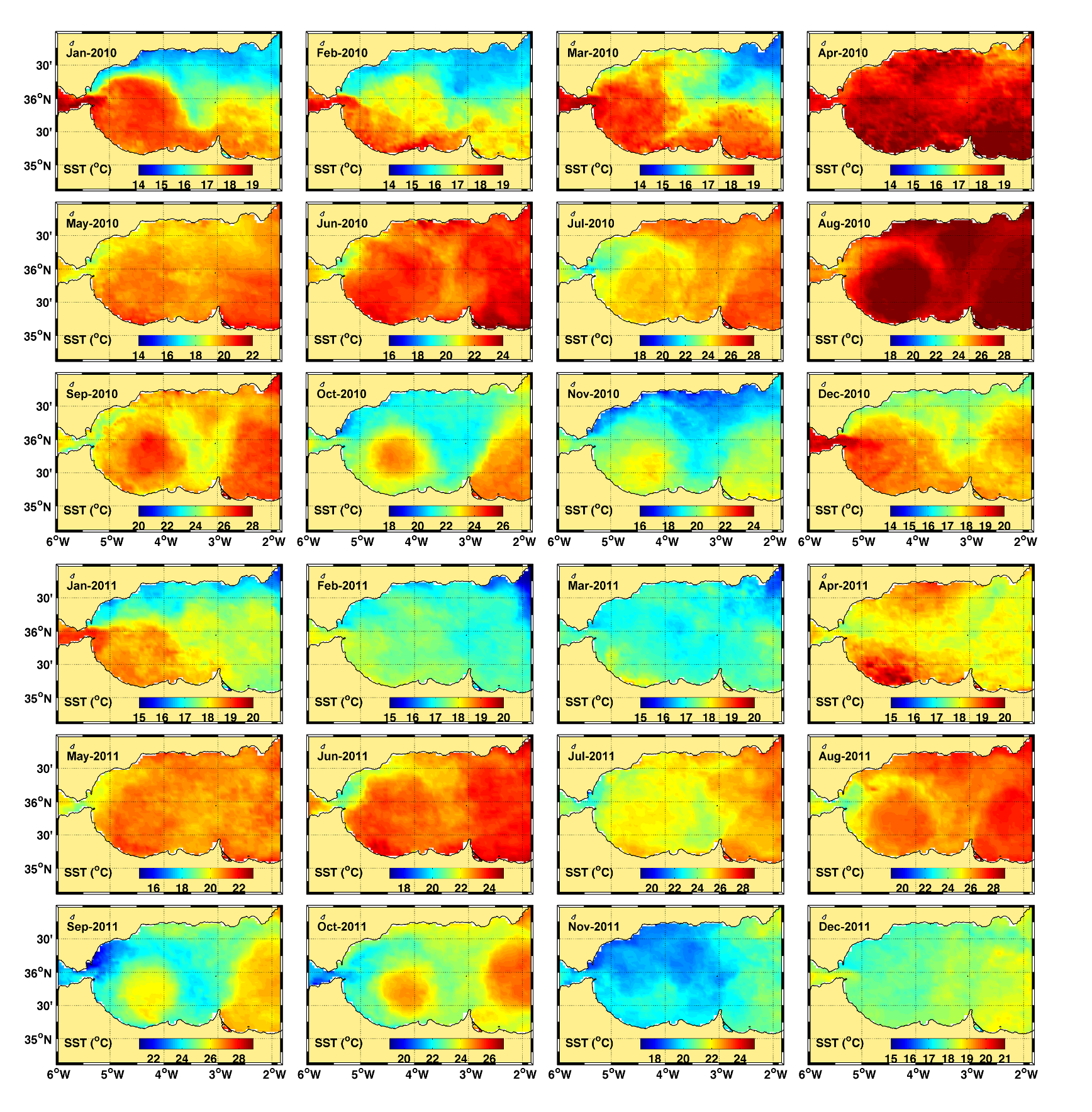

Supplement: Figure S12 — Monthly composites of Sea Surface Temperature (SST, in ) between 2010 and 2011. (TIFF) [file pone.0055523.s012.tiff]

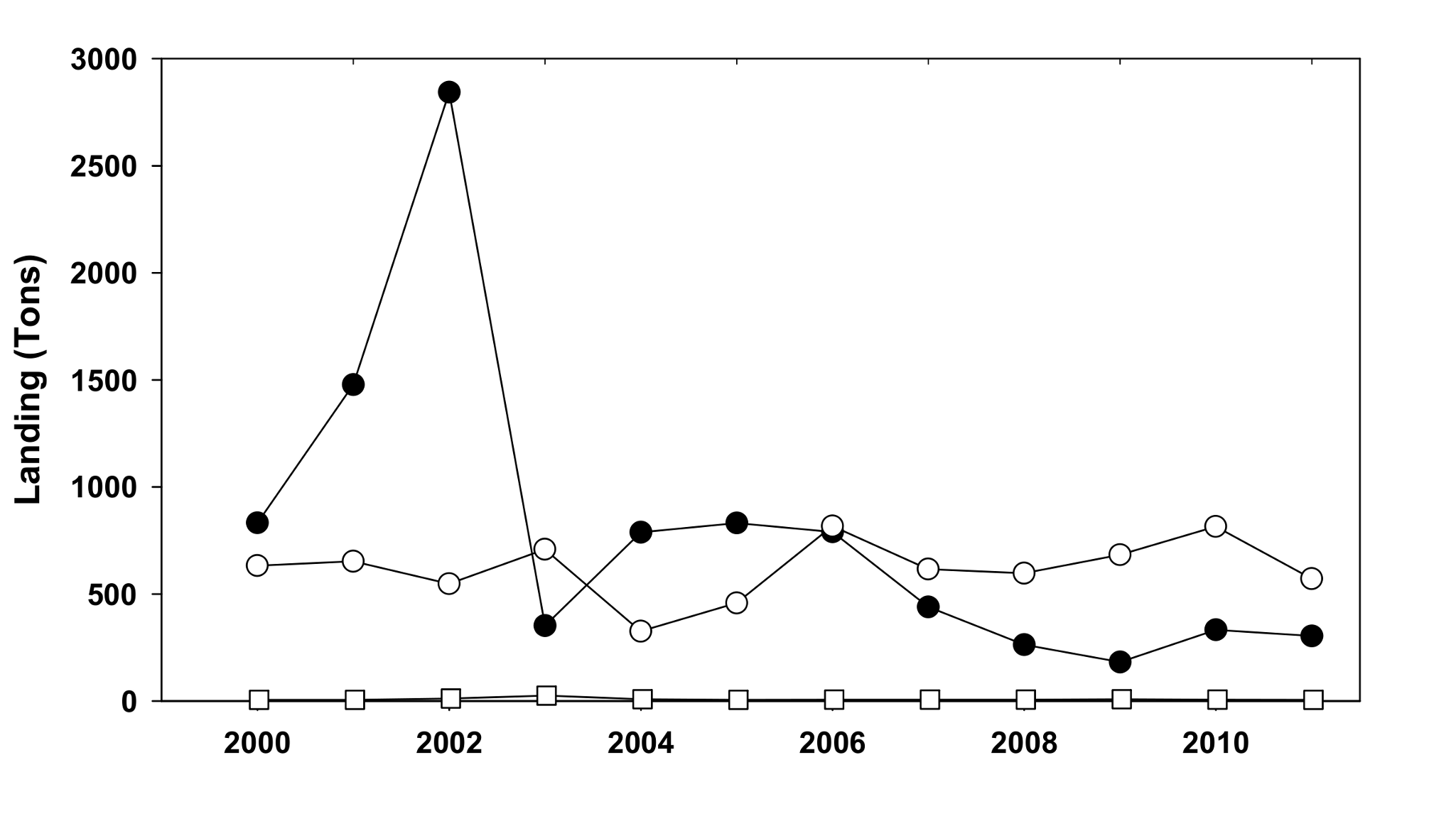

Supplement: Figure S13 — Yearly catches of small pelagic fish in the NW Alboran Sea during the last decade (data only reliable since 2000). Source: Andalusian Regional Government IDAPES database (http://www.juntadeandalucia.es/agriculturaypesca/idapes/). Black and white circle are anchovy and sardine respectively while the white square is mackerel. (TIFF) [file pone.0055523.s013.tiff]

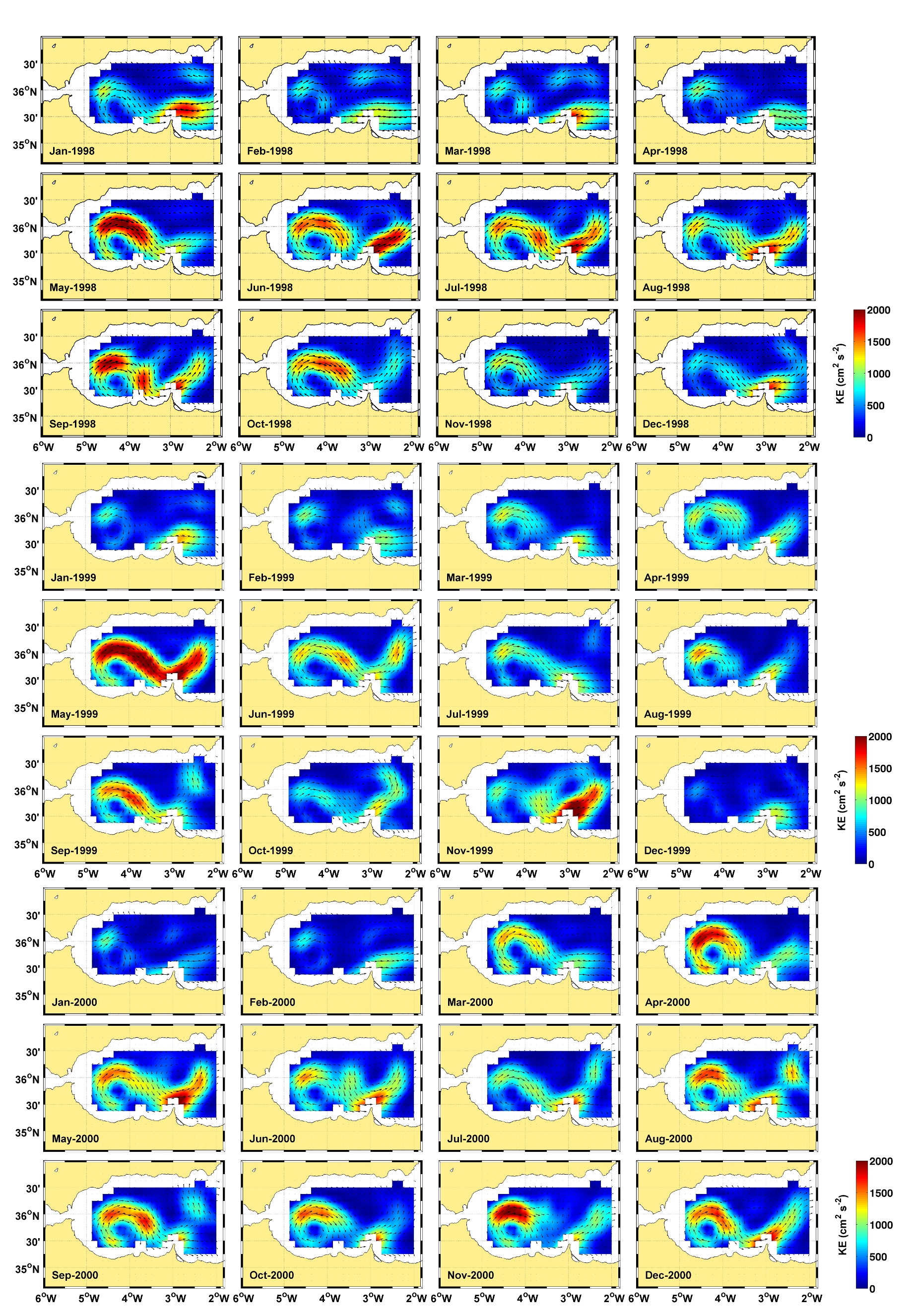

Supplement: Figure S14 — Monthly composites of geostrophic circulation and kinetic energy (KE, in ) derived from altimetry between 1998 and 2000. (TIFF) [file pone.0055523.s014.tiff]

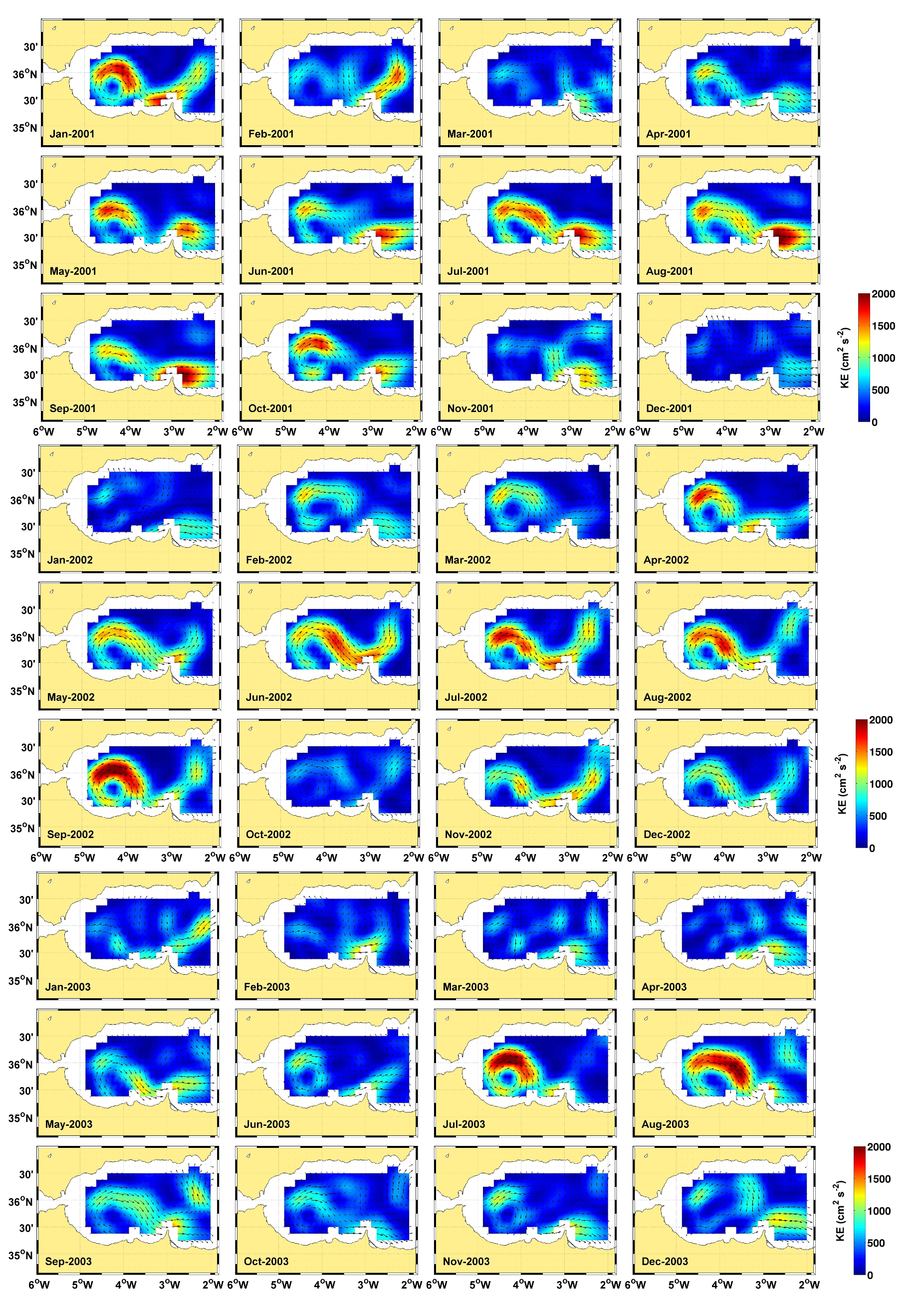

Supplement: Figure S15 — Monthly composites of geostrophic circulation and kinetic energy (KE, in ) derived from altimetry between 2001 and 2003. (TIFF) [file pone.0055523.s015.tiff]

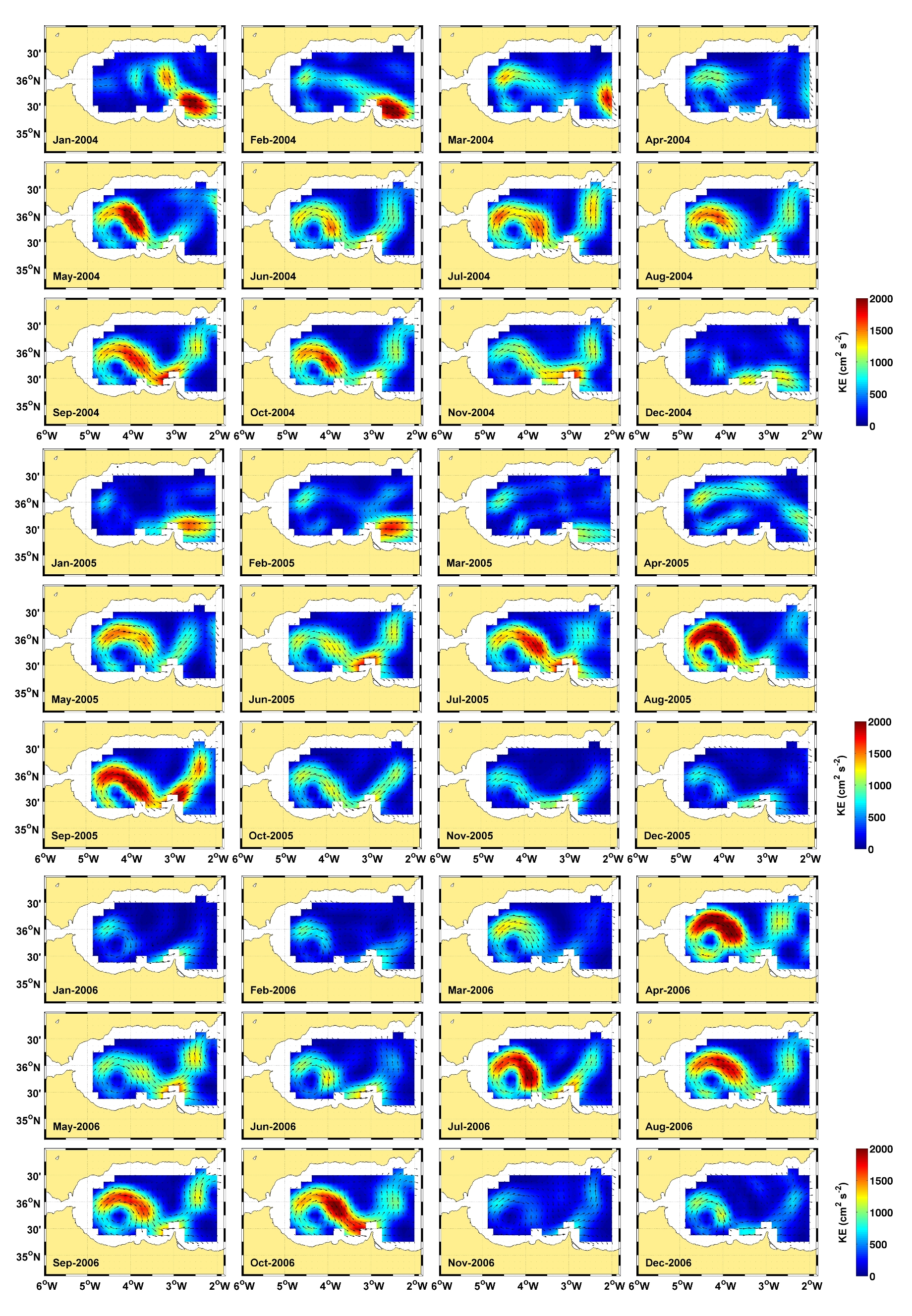

Supplement: Figure S16 — Monthly composites of geostrophic circulation and kinetic energy (KE, in ) derived from altimetry between 2004 and 2006. (TIFF) [file pone.0055523.s016.tiff]

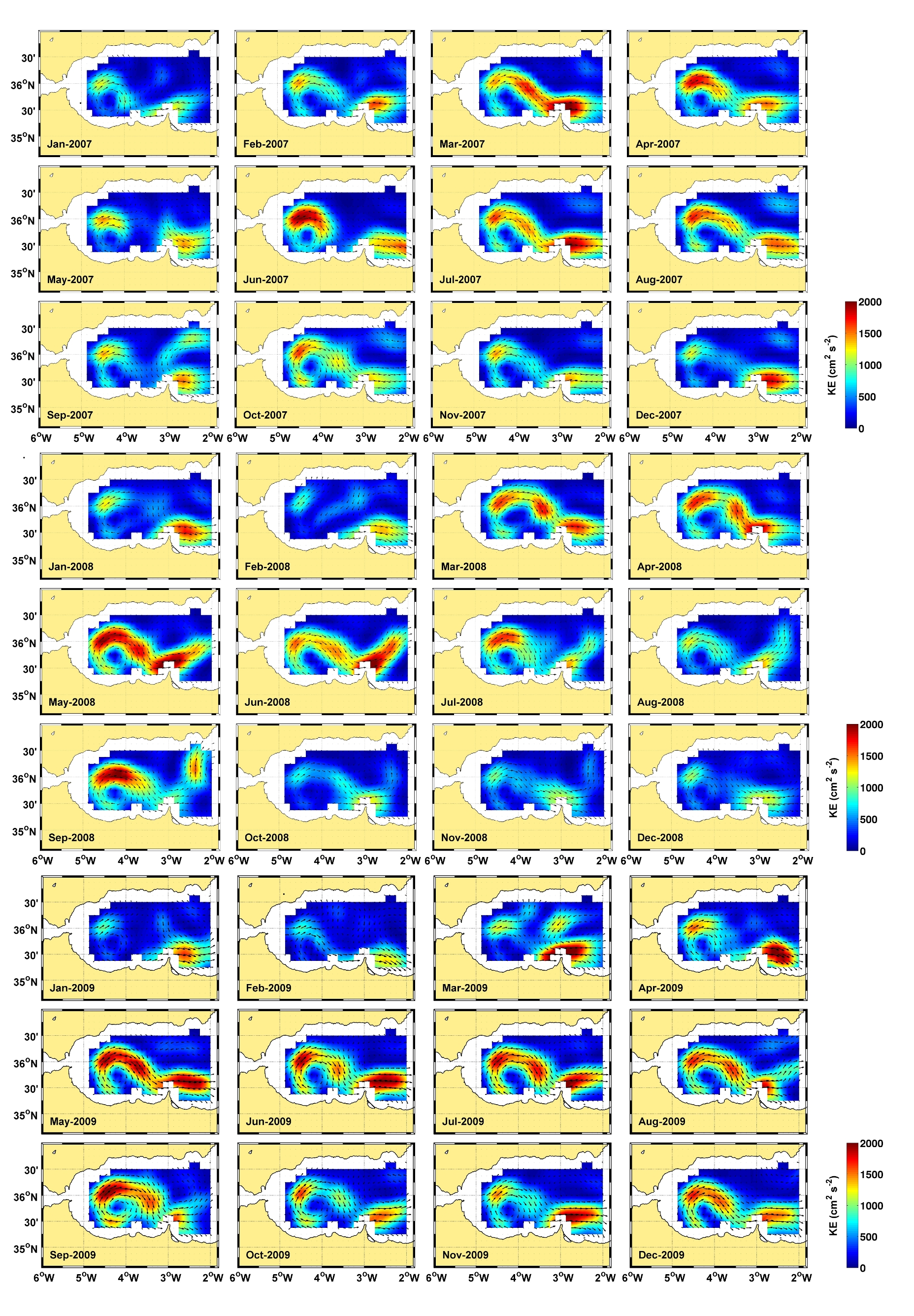

Supplement: Figure S17 — Monthly composites of geostrophic circulation and kinetic energy (KE, in ) derived from altimetry between 2007 and 2009. (TIFF) [file pone.0055523.s017.tiff]

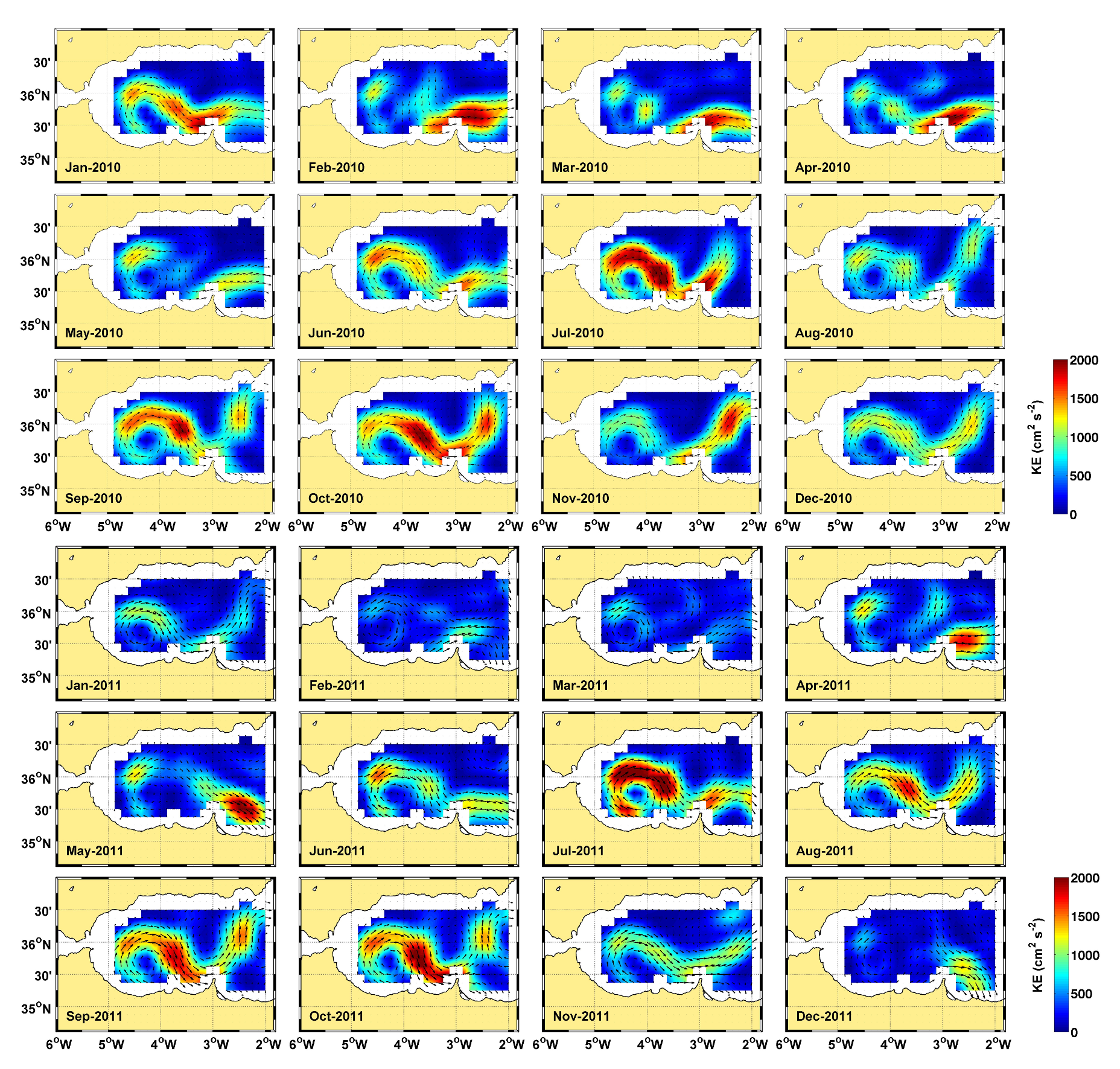

Supplement: Figure S18 — Monthly composites of geostrophic circulation and kinetic energy (KE, in ) derived from altimetry between 2010 and 2011. (TIFF) [file pone.0055523.s018.tiff]
